# Identification of a modular super-enhancer in murine retinal development

**DOI:** 10.1038/s41467-021-27924-y

**Published:** 2022-01-11

**Authors:** Victoria Honnell, Jackie L. Norrie, Anand G. Patel, Cody Ramirez, Jiakun Zhang, Yu-Hsuan Lai, Shibiao Wan, Michael A. Dyer

**Affiliations:** 1 Department of Developmental Neurobiology, St. Jude Children’s Research Hospital, Memphis, TN 38105 USA; 2 Graduate School of Biomedical Sciences, St. Jude Children’s Research Hospital, Memphis, TN 38105 USA; 3 Center for Applied Bioinformatics, St. Jude Children’s Research Hospital, Memphis, TN 38105 USA

**Keywords:** Developmental neurogenesis, Epigenetics in the nervous system, Development, Retina, Gene regulation

## Abstract

Super-enhancers are expansive regions of genomic DNA comprised of multiple putative enhancers that contribute to the dynamic gene expression patterns during development. This is particularly important in neurogenesis because many essential transcription factors have complex developmental stage– and cell–type specific expression patterns across the central nervous system. In the developing retina, Vsx2 is expressed in retinal progenitor cells and is maintained in differentiated bipolar neurons and Müller glia. A single super-enhancer controls this complex and dynamic pattern of expression. Here we show that deletion of one region disrupts retinal progenitor cell proliferation but does not affect cell fate specification. The deletion of another region has no effect on retinal progenitor cell proliferation but instead leads to a complete loss of bipolar neurons. This prototypical super-enhancer may serve as a model for dissecting the complex gene expression patterns for neurogenic transcription factors during development. Moreover, it provides a unique opportunity to alter expression of individual transcription factors in particular cell types at specific stages of development. This provides a deeper understanding of function that cannot be achieved with traditional knockout mouse approaches.

## Abstract (web summary)

Super-enhancers are regions of genomic DNA comprised of multiple putative enhancers that contribute to dynamic gene expression patterns during development. Here the authors identify a modular super-enhancer in murine retinal development and show that distinct modules are responsible for retinal progenitor cell proliferation during early and bipolar neuron genesis during late retinal development.

## Introduction

The developing retina must express thousands of genes in a precise spatiotemporal order for proper cell-fate determination^1^. While some genes, such as those regulated by Rb1, have simple transcriptional regulation at the transcription start site, others rely on enhancers that may be hundreds of kilobases away^2,3^. Looping out of the intervening chromatin and higher-order chromatin structures are believed to be important in developmental stage-specific promoter-enhancer interactions^4,5^. Indeed, in the case of *Sox2* the enhancer is not only physically separated from the promoter when the gene is inactive but it is sequestered in a different nuclear compartment^6,7^. Advances in high-throughput sequencing assays such as chromatin immunoprecipitation sequencing (ChIP-seq) has allowed for tens of thousands of putative enhancers to be identified in the developing murine and human retina^8–10^. However, few of these enhancers have been functionally analyzed in vivo^11^. One challenge is determining which enhancers are essential in retinogenesis and which genes they regulate. Another challenge is determining how genes with complex expression patterns in multiple cell types at different stages of development are regulated.

Super-enhancers (SEs) are large regulatory elements containing clusters of smaller transcriptional enhancers believed to define cell identity and cell fate specification across development^11–15^. Originally, SEs were identified in ESCs and were differentiated from regular enhancers through enrichment of Med1, OSN, H3K27ac, and H3K4me1, and DNaseI hypersensitivity regions^14^. Efforts to dissect SEs by genetic manipulation suggest that SEs are comprised of both necessary and dispensable elements^16–21^. Whether constituent enhancers comprising SEs are responsible for gene expression, or whether SEs act as a single functional regulatory unit is still debated in the field.

Many central nervous system-specific transcription factors have complex expression patterns since they are expressed in multiple cell types across multiple stages of development^22–24^. This makes uncoupling early-stage effects from late-stage effects difficult in gene knockout mice. For example, Pax6 expression is required for both brain and eye formation early in development, is downregulated as neurons differentiate, and is maintained in a subset of differentiated cell types in the adult^25,26^. Since a *Pax6* knockout animal model is embryonic lethal, studying its late-stage effects in postmitotic neurons is difficult even with conditional knockout approaches. The same is true for many of the transcription factors that are essential for neurogenesis.

The *Vsx2* gene encodes a homeodomain containing transcription factor that is expressed in several cell types across multiple stages of eye development^27,28^. Vsx2 is found in the majority of proliferating retinal progenitor cells (RPCs) during development as well as differentiated bipolar cells, and Müller glia in the adult retina^27–30^. Mutations in human and mouse *VSX2/Vsx2* lead to microphthalmia because it is required for retinal progenitor cell proliferation^31–34^. Bipolar cells have not been detected in the *Vsx2* mutant retina, but it is not clear if this is due to a direct role of Vsx2 in bipolar cell fate specification or a secondary consequence of the defects in RPC proliferation^31,33^.

We previously identified a super-enhancer upstream of the *Vsx2* gene^35^ and computational methods^36^ suggested it was a core regulatory circuit SE (CRC-SE). That is, the *Vsx2* SE has a Vsx2 binding site and may regulate its own expression. Deletion of a portion of the *Vsx2* SE including the Vsx2 binding site led to a complete absence of bipolar neurons^35^. Importantly, the region we deleted contained a much smaller element that had been previously shown to confer bipolar-specific expression by square wave electroporation in vivo^37^. In a more recent study, that bipolar-specific element was deleted using CRISPR/Cas9 by in vivo square wave electroporation leading to a reduction in bipolar neurons^38^. Vsx2 expression was maintained in Müller glia of *Vsx2-SE* mice and there was no evidence of microphthalmia suggesting that the retinal progenitor expression of Vsx2 was not altered.

In the present study, we further refine the domains of the *Vsx2* CRC-SE and determine if Vsx2 binding is required for its function by generating eight new mouse strains lacking different regions of the *Vsx2* SE. We show that the previously identified evolutionarily conserved Vsx2 binding sites are not required for function and we identify distinct subdomains or modules of the larger *Vsx2* SE that are required for retinal progenitor cell expression and bipolar neuron expression. Single-cell RNA-seq combined with single-cell ATAC-seq was particularly useful for identifying these distinct domains, but in vivo validation was essential for establishing their function. It is possible that SEs and/or CRC-SEs associated with neurogenic transcription factors that have complex spatial and temporal patterns of expression may be modular with respect to the patterns of expression. This is in contrast to SEs and/or CRC-SEs where the individual elements are simply additive, but do not confer temporal or spatial specificity^15^.

## Results

### Bipolar cells switch fate in the *Vsx2-SE*^*∆/∆*^ retina

**Fig. 1:**
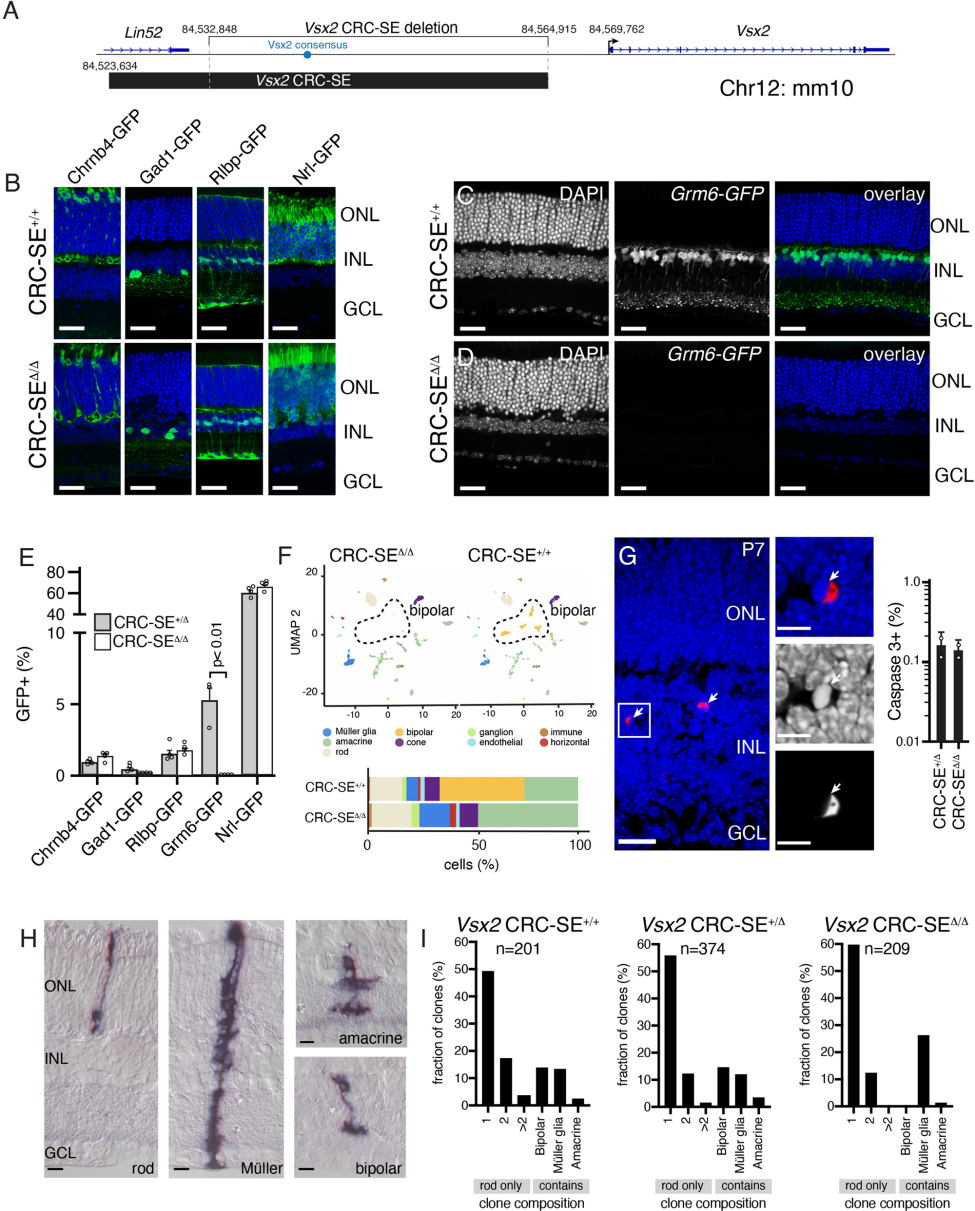
Cell-type specification in the Vsx2 CRC-SE deletion retina. **A** Drawing of the original Vsx2 CRC-SE identified by H3K27Ac ChIP-seq (black bar) and the original Vsx2 CRC-SE deletion with coordinates in mm10. **B** The Vsx2 CRC-SE deletion strain was crossed to GFP reporter strains for different retinal cell types as indicated. Micrographs of representative sections for wild type (upper) and knockout (lower panel) are shown. **C**, **D** Micrograph of Grm6-GFP expression in ON bipolar cells of wild type (**C**) and Vsx2 SE knockout. DAPI staining is blue and GFP expression is green. **E** Bar plot showing mean with SEM for FACS analysis of the proportion of GFP+ cells for each strain shown in **B**–**D** (*n* = 4 retinae from two mice, unpaired two-sided *t*-test, *p* = 0.0016). **F** tSNE plot of scRNA-seq of rod depleted retinae showing each individual cell type for each genotype. The bipolar cluster is outlined. **G** Micrograph of DAPI (blue) and activated caspase three (red) immunostaining of retinal sections at P7 for the Vsx2 SE knockout retina. A bar plot showing the scoring from two independent experiments with mean and standard deviation adjacent to the micrographs. **H** Micrographs of alkaline phosphatase stained clones of cells showing rods, Müller, amacrine, and bipolar neurons based on laminar position and morphology. **I** Bar plot showing the scoring of clone composition for each genotype and the total number of clones. There is a loss of bipolar containing clones and a corresponding increase in rod and Müller glia containing clones (chi-squared test, *p* = 0.0053). Abbreviations: ONL, outer nuclear layer; INL, inner nuclear layer; GCL, ganglion cell layer; CRC, core regulatory circuit. Scale bars: **B**–**D**, 25 mm, **G**, **H**, 10 mm. Source data are provided as a Source Data file.

We have previously identified a 41 kb CRC-SE upstream of the *Vsx2* gene (Fig. 1A)^10^. Deletion of 32 kb of that SE (Fig. 1A) in vivo using CRISPR/Cas9 led to a complete loss of bipolar neurons based on immunostaining and RNA-seq^35^. To further elucidate the cell-type specific effects of the Vsx2-SE deletion, we crossed the *Vsx2-SE*^*∆/∆*^ mice to those with cell-type specific GFP reporter transgenes (Fig. 1B–D). The pattern of GFP expression in cones (*Chrnb4-GFP*), horizontal cells, and a subset of amacrine cells (*Gad1-GFP*), Müller glia (*Rlbp-GFP*), and rods (*Nrl-GFP*) was normal other than the laminar disruption that occurs due to the loss of bipolar cells (Fig. 1B). However, ON bipolar cells were completely absent in the *Vsx2-SE*^*∆/∆*^*; Grm6-GFP* mice^39–42^ (Fig. 1C, D, F). Quantitative scoring of the proportion of GFP + cells in each line by flow cytometry showed that there were no statistically significant changes in retinal cell types other than bipolar neurons (Fig. 1E). However, subtle differences may be obscured by the large proportion (~80%) of rods in the murine retina.

The reduction in bipolar neurons in the *Vsx2-SE*^*∆/∆*^ retinae raises the possibility that the cells fated to be bipolar neurons die during development or become other cell types born at the same stage of development (rods or Müller glia). To determine if bipolar neurons died during development, we performed immunostaining for activated Caspase 3 during the peak period of bipolar genesis (P3, P7) in wild type and *Vsx2-SE*^*∆/∆*^ retinae and scored the proportion of immunopositive cells. There was no observable difference in the proportion of apoptotic cells between wild type and *Vsx2-SE*^*∆/∆*^ retinae (Fig. 1G). Next, to determine if there is an increase in rods and/or Müller glia, we performed retroviral lineage analysis using an alkaline phosphatase replication-incompetent retrovirus (LIA-E, Supplemental Information). The retrovirus was injected into the subretinal space in P0 pups from a *Vsx2-SE*^*+/∆*^ intracross. After 3 weeks, the retinae were harvested, stained for alkaline phosphatase, and serially cryosectioned. Clones of cells from individual retinal progenitor cells were reconstructed from serial sections and scored for cell number and cell type by morphology and laminar position (Fig. 1H). After scoring, the mice were genotyped to determine if they were *Vsx2-SE*^*∆/∆*^*, Vsx2-SE*^*+/∆*^, or *Vsx2-SE*^*+/+*^. We found significant differences in cell type distribution according to genotype (*p* = 0.0053). Notably, bipolar cell containing clones were absent in the *Vsx2-SE*^*∆/∆*^ retinae while they were present in the *Vsx2-SE*^*+/∆*^ and *Vsx2-SE*^*+/+*^ retina. The absence of bipolar containing clones was largely filled by an increased relative prevalence of Müller glia or rod containing clones in the *Vsx2-SE*^*∆/∆*^ relative to the wild type and heterozygous littermates (Fig. 1I and Supplemental Information). This is consistent with the birthdates of late born cell types (rods, bipolar neurons, and Müller glia). Taken together, our data suggest that the bipolar neurons become rods and Müller glia in the *Vsx2-SE*^*∆/∆*^ retina.

### Retinal progenitor cell proliferation is normal in the *Vsx2-SE*^*∆/∆*^ retina

**Fig. 2:**
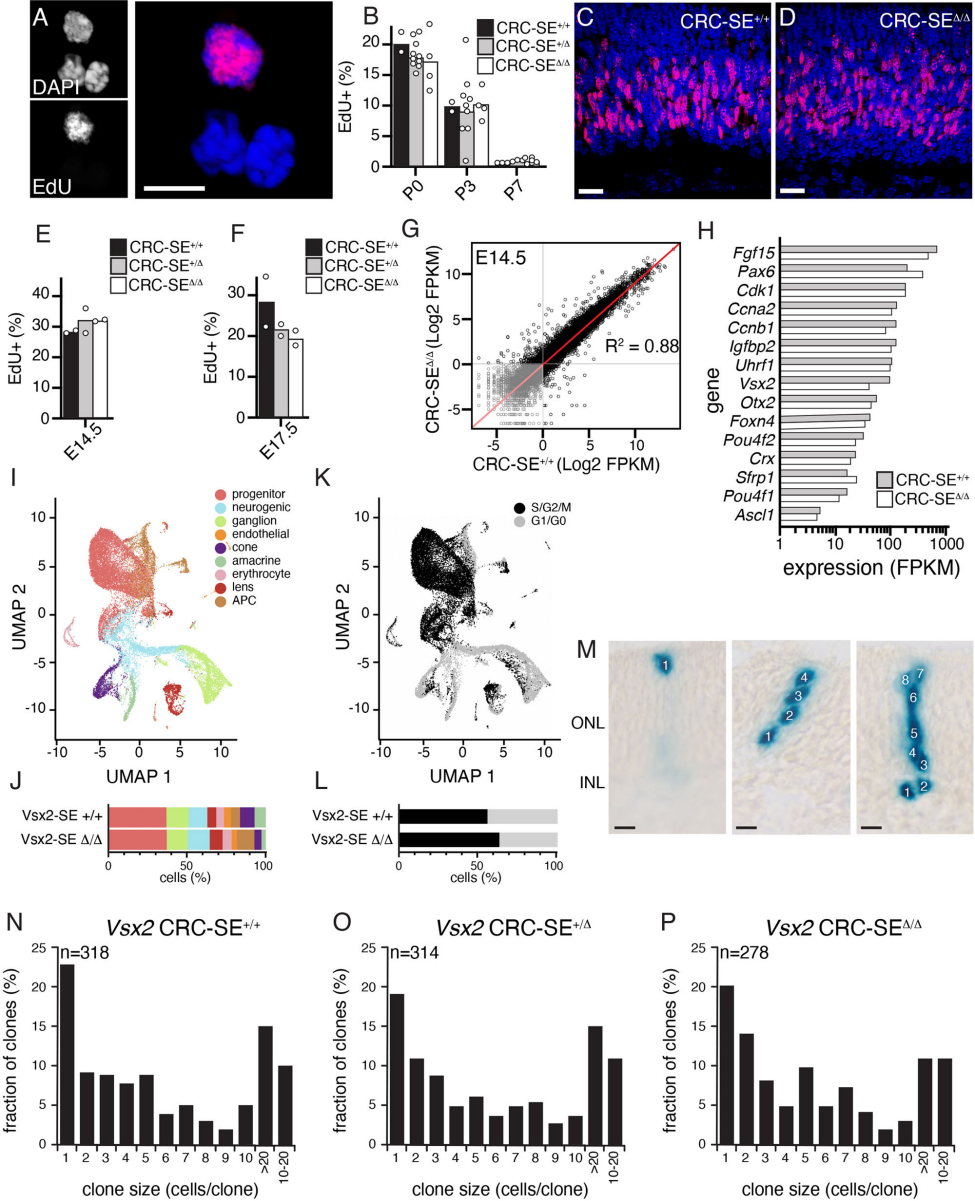
Proliferation is normal in the Vsx2 CRC-SE deletion retina. **A** Micrograph of dissociated P0 retinal cells showing EdU labeling (red) and DAPI (blue). **B** Bar plot showing the mean of retinae from littermates for dissociated cell scoring. At P0 *n* = 8, P3 *n* = 7, P7 *n* = 7. There is no observable difference across genotypes or stages. **C**, **D** Representative micrograph of sections of P0 retina showing DAPI (blue) and EdU (red). **E**, **F** Scoring of EdU+ cells from micrographs of sections. Bar plots display the mean of biological duplicates. There was no observable difference across genotypes. **G** Scatterplot of each gene in RNA-seq analysis in the CRC-SE deletion strain and wild-type mice at E14.5 with a correlation coefficient of 0.88. **H** Bar plot of expression in FPKM for representative retinal progenitor, cell cycle, and differentiation genes. **I**, **J** tSNE of scRNA-seq for wild type and Vsx2 CRC-SE deletion retina at E14.5 and corresponding stack bar plot. **K**, **L** Similar data as **I**, **J** but showing cell cycle distribution across retinal cell populations as indicated. **M** Representative micrograph of clones of cells from E14.5 retinal explants cultured for 2 weeks to score clone size. **N**–**P** Scoring of clone size distribution across the genotypes from littermates of a heterozygous intercross (Kruskal–Wallis test, number of clones *p* = 0.784, median number of cells per clone *p* = 0.6634). Abbreviations: FPKM, fragments per kilobase per million reads. Scale bars: **A**, 5 mm; **C**, **D**, and **M**, 10 mm. Source data are provided as a Source Data file.

To determine if there is any perturbation in retinal progenitor cell proliferation as a result of the super-enhancer deletion, we performed EdU labeling. *Vsx2-SE*^*+/∆*^ mice were intercrossed to generate *Vsx2-SE*^*∆/∆*^*, Vsx2-SE*^*+/∆*^, and *Vsx2-SE*^*+/+*^ littermates. Animals were injected with EdU and 1 h later the retinae were harvested, stained for EdU and scored (Supplemental Information). There was no observed difference in the proportion of EdU+ cells at E14.5, E17.5, P0, P3, or P7 (Fig. 2A–F). RNA-seq on E14.5 *Vsx2-SE*^*∆/∆*^and *Vsx2-SE*^*+/+*^ retinae showed no observable difference in expression of cell cycle, retinal progenitor, or retinal development genes (Fig. 2G, H). We confirmed those results at single-cell resolution by performing scRNA-seq on E14.5 *Vsx2-SE*^*∆/∆*^ and *Vsx2-SE*^*+/+*^ retinae using the analysis method described above for Fig. 1 (Fig. 2I–L). Finally, to confirm that the proliferation of individual retinal progenitor cells was similar in *Vsx2-SE*^*∆/∆*^*, Vsx2-SE*^*+/∆*^, and *Vsx2-SE*^*+/+*^ retinae, we performed clonal analysis using a replication incompetent retrovirus that expresses nuclear β-galactosidase (NIN-E, Supplemental Information). Briefly, retinae from E14.5 embryos were harvested from a *Vsx2-SE*^*+/∆*^ intercross and cultured as explants. They were infected with the NIN-E retrovirus, cultured for 14 days, genotyped and stained for β-galactosidase expression (Fig. 2M). Individual clones of cells derived from single infected retinal progenitor cells were reconstructed from serial sections and scored for cell number. There are no statistically significant differences in the number of clones (*p* = 0.784) or median number of cells per clone (*p* = 0.663) according to genotype (Fig. 2N, P). Taken together, these data suggest that deletion of the 3' 32 kb portion of the 41 kb *Vsx2-SE* affects the expression of Vsx2 in bipolar neurons but not Müller glia or retinal progenitor cells. There is no defect in retinal progenitor cell proliferation and the cells that would normally become bipolar neurons now develop as rod photoreceptors and Müller glia.

### The Vsx2-SE contains multiple evolutionarily conserved regions

**Fig. 3:**
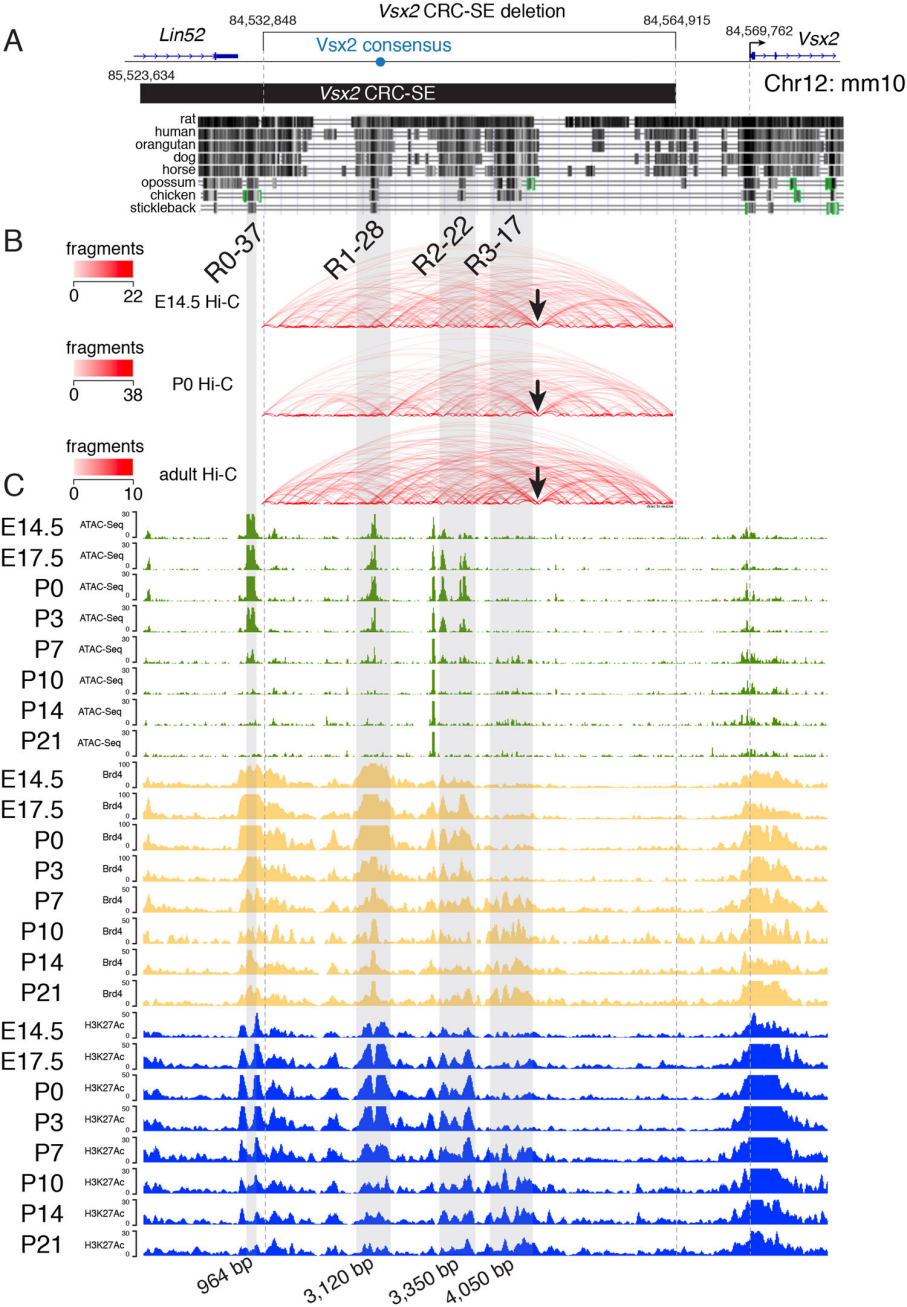
Chromatin landscape of Vsx2 CRC-SE. **A** Evolutionary conservation of Vsx2 CRC-SE regions. **B** Hi-C interaction map at E14.5, P0 and adult showing a separation just downstream of the evolutionarily conserved regions (arrow). **C** Representative ATAC-seq and ChIP-seq for Brd4 and H3K27Ac across developmental stages for each region.

As a first step to identify the module(s) of the *Vsx2* SE required for Vsx2 expression in bipolar neurons, we aligned the evolutionarily conserved regions with our previous ChIP-seq and ATAC-seq data from the developing mouse retina^35^. We identified three evolutionarily conserved regions (R1-28, R2-22, and R3-17) that are 28, 22, and 17 kb upstream of the *Vsx2* transcription start site and contained within the original 32 kb *Vsx2-SE* deletion (Fig. 3A). An additional evolutionarily conserved region (R0-37) was identified 37 kb upstream of the *Vsx2* transcription start site. R0-37 was not part of the original 32 kb deletion but is contained in the 41 kb Vsx2 SE computationally defined from our original H3K27Ac ChIP-seq data (Fig. 3A). This region overlaps with an evolutionarily conserved sequence from the human genome that was previously shown to be sufficient for driving reporter gene expression in retinal progenitor cells in chick and mouse retina^30^. R0-37 and R1-28 have ATAC-seq, Hi-C, and ChIP-seq profiles consistent with retinal progenitor cell expression while R3-17 is consistent with bipolar and/or Müller glial cell expression in adult retina (Fig. 3B, C)^10,35^. Importantly, R3-17 overlaps with a genomic segment that has been previously shown by the Cepko research group to be sufficient for bipolar expression of a transgene in vivo^28,37,43^. The Hi-C data suggests that there is a looping interaction between the conserved elements and the *Vsx2* promoter in E14.5, P0, and adult retina (Fig. 3B).

### The evolutionarily conserved regions have cell-type specific accessibility and activity

**Fig. 4:**
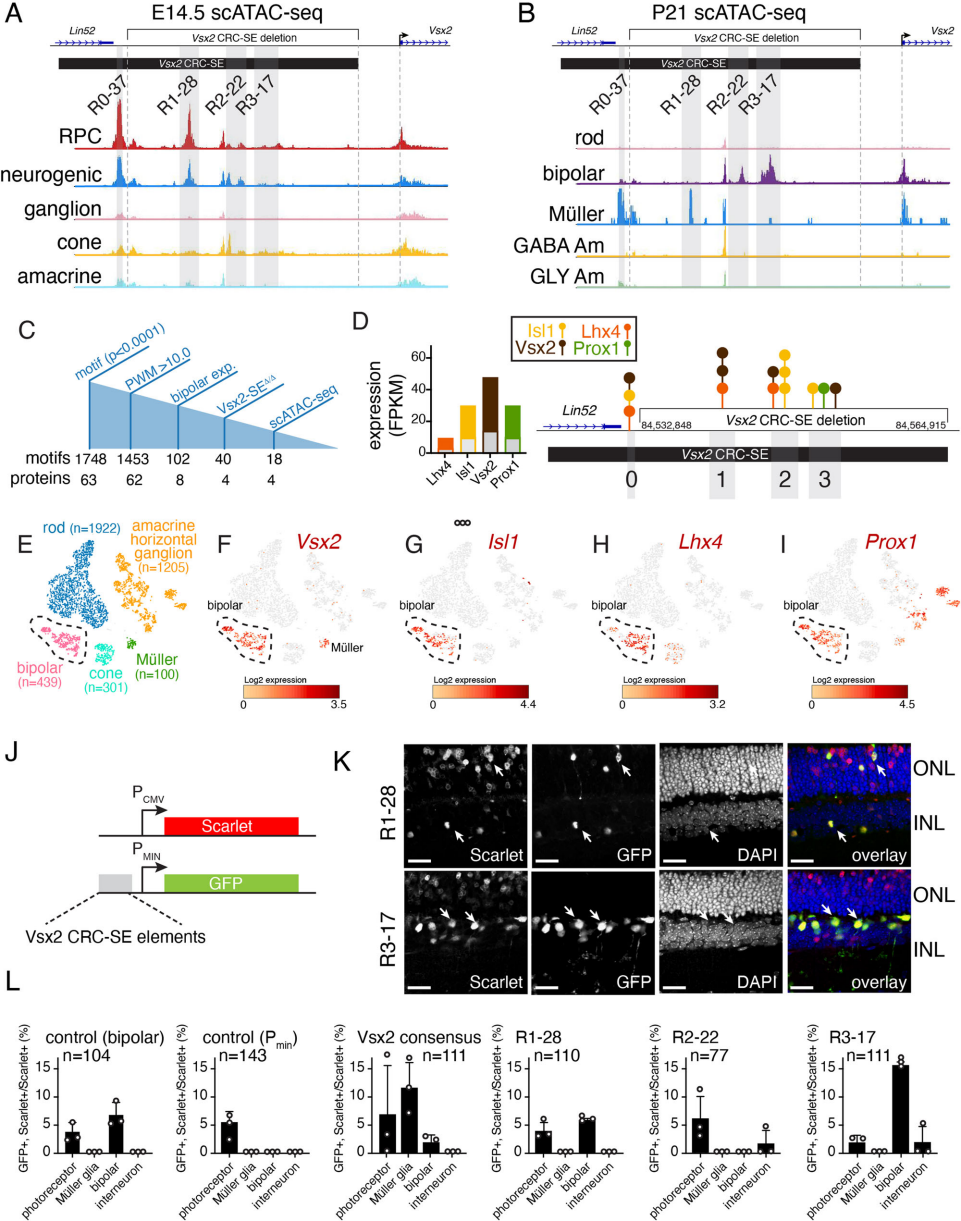
Transcription factor binding sites in each sub-region of the Vsx2 CRC-SE. **A**, **B** scATAC-seq for E14.5 and adult retina showing bipolar-specific chromatin accessibility and retinal progenitor/Müller glia specific accessible regions. **C** Drawing of the motif analysis in the Vsx2 CRC-SE. **D** Overlapping bar plot of FPKM for each of the four bipolar-specific transcription factors with binding sites in the Vsx2 CRC-SE. The expression (FPKM) from bulk RNA-seq is shown with colored bars and the corresponding expression in the Vsx2 SE knockout is shown with the gray bars on top of the colored bars. Each was reduced by at least 2-fold in the Vsx2 SE knockout. The map of the binding sites for the four bipolar-specific transcription factors in the Vsx2 CRC-SE that overlap with the evolutionarily conserved regions is shown to the right. **E**–**I** Expression of the transcription factors in the normal retinal cell types. **J** Drawing of plasmids used for reporter assays for in vivo square wave electroporation at P0 in mice and harvested at P21. A minimal promoter (P_MIN_) that is not sufficient for high-level expression is upstream of GFP and a strong constitutive promoter (P_CMV_) is used for the scarlet reporter. **K** Micrographs of GFP (green) and scarlet (red) expression at P21 from square wave electroporation of P0 mice of reporters with R1-28 or R3-17 subcloned upstream. Arrows indicate bipolar neuron expression for the R3-17 fragment and rods and Müller glia for the R1-28 fragment. **L** Bar plot showing mean and standard deviation of three biological replicates for each reporter construct. The positive control has a previously identified bipolar-specific element, the negative control is just the minimal promoter with very little expression. Abbreviations: RPC, retinal progenitor cell. Scale bars: **K**, 25 mm. Source data are provided as a Source Data file.

To further refine the cell-type specificity of evolutionarily conserved regions with open chromatin, we performed scATAC-seq on E14.5 and adult retinae. Cell types were identified based on the open chromatin at promoters for cell-type specific genes (Supplemental Information). Consistent with the data in Fig. 3, chromatin in regions 0 and 1 were most accessible in retinal progenitor cells (Fig. 4A). Interestingly, those regions were also in an open chromatin state in Müller glia of the adult retina (Fig. 4B). This is in contrast to R3-17 which has scATAC-seq peaks specifically in bipolar neurons of the adult retina (Fig. 4B). Next, we identified putative transcription factors that may contribute to Vsx2 gene expression by identifying consensus binding sites. There are 1,748 predicted motifs for 63 different transcription factors (*p*-value < 0.0001) and 1,453 motifs (62 transcription factors) have a positional weight matrix (PWM) > 10.0 (Fig. 4C). Only eight of the 62 transcription factors (*Dbp, Hlf, Otx2, Zfhx3, Prox1, Vsx2, Isl1*, and *Lhx4*) are expressed in bipolar neurons (102 motifs) and four (*Isl1, Lhx4, Prox1*, and *Vsx2*) have greater than 2-fold reduction in expression in the *Vsx2-SE*^*∆/∆*^ retinae (Fig. 4C–I). There are 40 motifs for Isl1, Lhx4, Vsx2, and Prox1 across the entire Vsx2-SE but only 18 overlap with the scATAC-seq peaks in the evolutionarily conserved regions (Fig. 4B and Dataset S1). Vsx2 is expressed in bipolar neurons and Müller glia, Isl1, and Lhx4 are expressed primarily in bipolar neurons and Prox1 is expressed in bipolar neurons and a subset of amacrine cells (Fig. 4E–I). We performed ChIP-seq for Isl1, Prox1, and Lhx4 and compared those data to our previous results for Vsx2. Lhx4 ChIP-seq failed quality control and Vsx2, Isl1, and Prox1 all had ChIP-seq peaks at the consensus binding sites predicted from our analysis (Fig. 4D and Fig. S1A–C). While there was a one-to-one correspondence for ChIP-seq peaks and the predicted binding sites for Vsx2 and Prox1, there were several predicted Isl1 binding sites that did not have corresponding ChIP-seq peaks. This highlights the importance of validating predicted binding sites with ChIP-seq.

In order to determine if regions 1–3 were sufficient to drive expression in bipolar neurons and/or Müller glia, we subcloned them into a GFP reporter plasmid with a minimal promoter that has been described previously^37^ (Fig. 4J). The individual R1-28, R2-22 and R3-17 reporter plasmids were separately co-electroporated in vivo at P0 with a constitutive Scarlet reporter to label all electroporated cells^44^. We also included a reporter plasmid with the Vsx2 consensus binding sites within R1-28. As a positive control for cell-type specific expression we used a previously identified bipolar-specific regulatory element^37^. We scored proportion of GFP + ,Scarlet + /Scarlet+ cells for each cell type for each reporter plasmid at P21 as done previously^45,46^. R2-22 had little activity in this assay and the Vsx2 binding sites conferred Müller glial cell expression (Fig. 4K, L). R1-28 had significantly more bipolar cell expression than the negative control (*p* = 0.0018) but R3-17 had significantly more bipolar cell activity than R1-28 (*p* = 0.0028). As further confirmation, we performed in vivo square wave electroporation of a plasmid that confers bipolar neuron specific expression of CRISPR and gRNAs for the Vsx2 gene or R3-17 enhancer element (see Supplemental Materials and Methods). For both sets of gRNAs, there was a reduction in bipolar neurons in the adult retina (Fig. S1E). Taken together, the scATAC-seq data and in vivo square wave electroporation data are consistent with the major bipolar-specific regulatory element residing in R3-17.

To test the activity of regions 1–3 in proliferating retinal progenitor cells, we performed ex vivo square wave electroporation on E14.5 retinae and in vivo square wave electroporation on P0 retinae as described above. We also included R0-37 because of the strong scATAC-seq peak in retinal progenitor cells. Forty-eight hours after electroporation, EdU was added to the explant culture medium (E14.5 electroporation) or administered to the pup (P0 electroporation) by intraperitoneal injection and retinae were harvested 1 h later. We scored the proportion of EdU+ , GFP+ , Scarlet+ /Scarlet+ for each plasmid in biologic triplicate. R0-37 showed the highest level of expression in proliferating retinal progenitor cells (Fig. S1F, G).

### The Vsx2 SE contains distinct developmental—and cell type—specific modules

**Fig. 5:**
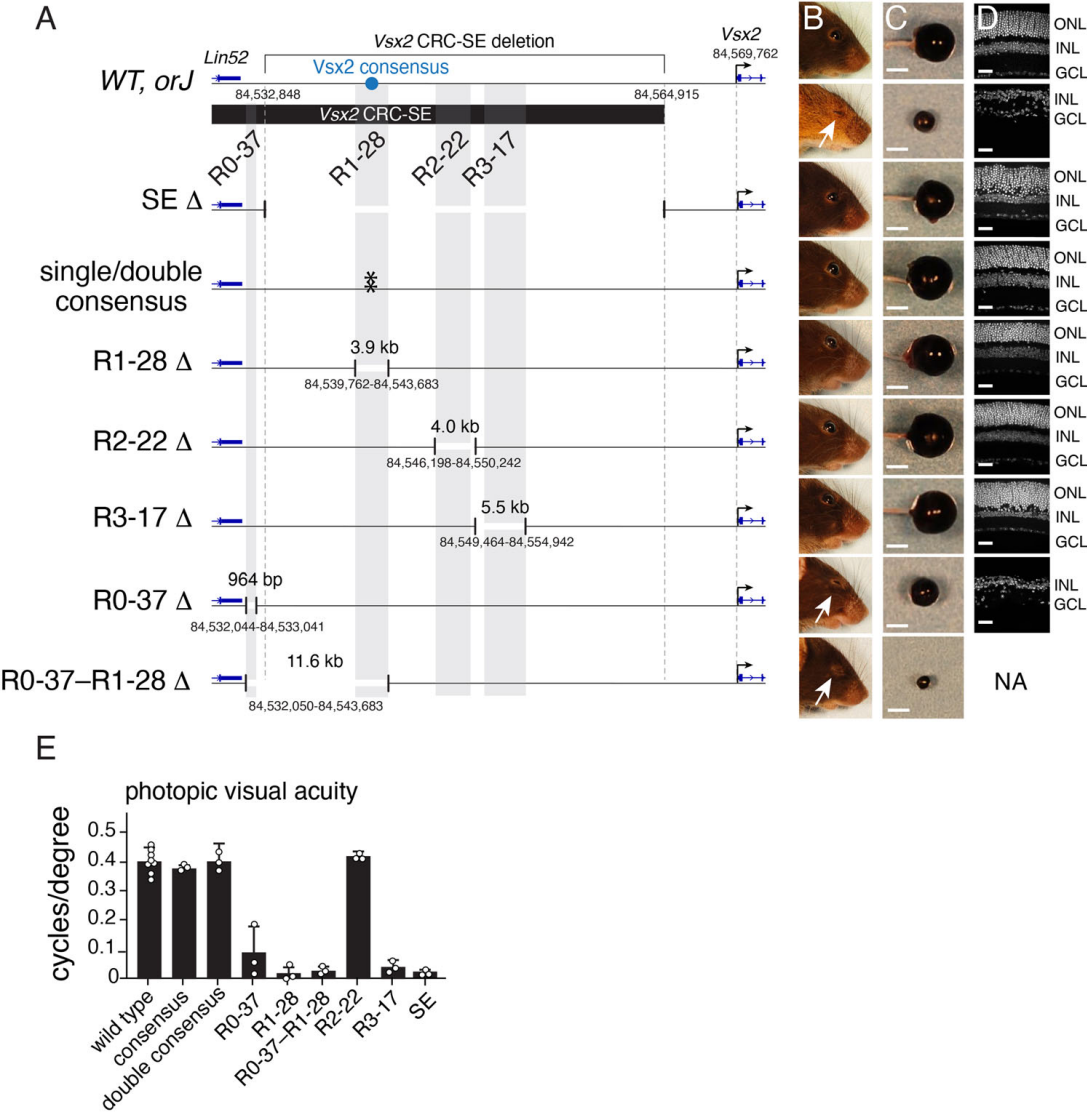
Analysis of mouse strains with deletions of evolutionarily conserved domains of the Vsx2 SE. **A** Drawing of the eight strains with deletions made using CRISPR-Cas9. There were three separate lines used for the consensus that had each site mutated individually and the combination of the two sites mutated. **B** Photograph of adult mice with each homozygous deletion and the *orJ* mouse strain as a reference for microphthalmia. **C** Photograph of eyes for each strain showing microphthalmia for the *orJ*, R0-37, and R0-37–R1-28 strains. **D** DAPI stained micrographs of retina from each strain. **E** Bar plot of mean with SEM of photopic vision (cycles/degree). *n* = 3 biologically independent mice for each deletion mouse strain. *n* = 8 biologically independent animals for wild-type mice. Abbreviations: ONL, outer nuclear layer; INL, inner nuclear layer; GCL, ganglion cell layer; Scale bars: 2.5 mm. Source data are provided as a Source Data file.

The developmental—and cell type–specific chromatin profiling combined with the in vivo electroporation data suggest that the 41 kb Vsx2 SE is bifunctional with R0-37 and R1-28 being active in retinal progenitor cells and Müller glia and R3-17 being active in bipolar neurons. We used CRISPR-Cas9 to delete each conserved region and the Vsx2 binding sites in mice (Fig. 5A). There are two adjacent Vsx2 consensus binding sites in R1-28 that were deleted individually and in combination (Fig. 5A). We also deleted the combination of R0-37 through R1-28 to determine if they function together in retinal progenitor cells. In total, eight new mouse lines were analyzed. Multiple independent founder lines were generated for each deletion and they were outcrossed to wild type (C57BL/6 J) mice before intracrossing individual sublines for each deletion. We compared these *Vsx2* SE deletion strains to the mouse strain (*orJ*) that has a spontaneous mutation in the *Vsx2* gene and microphthalmia^31,34^. The R0-37 mice had microphthalmia (Fig. 5B, C) and the retinae were hypocellular (Fig. 5D). The phenotype was not as severe as that observed in the *orJ* mice (Fig. 5B–D). The R1-28 mice had a normal size eye but the retina was approximately half the thickness of a wild-type retina (Fig. 5B–D). The combined deletion of R0-37 through R1-28 led to microphthalmia and the eyes were smaller than either single deletion alone (Fig. 5B–D). Indeed, the eyes were so small that we were unable to isolate sufficient retinae from the eyes for molecular profiling or histology (Fig. 5D). Visual acuity measurements under scotopic conditions were consistent with the microphthalmia (R0-37 and R0-37–R1-28), reduction in retinal thickness (R1-28) and loss of bipolar neurons (R3-17) (Fig. 5E). The mutations in the Vsx2 consensus binding sites and deletion of R2-22 had no effect on visual acuity or retinal development (Fig. 5A–E).

**Fig. 6:**
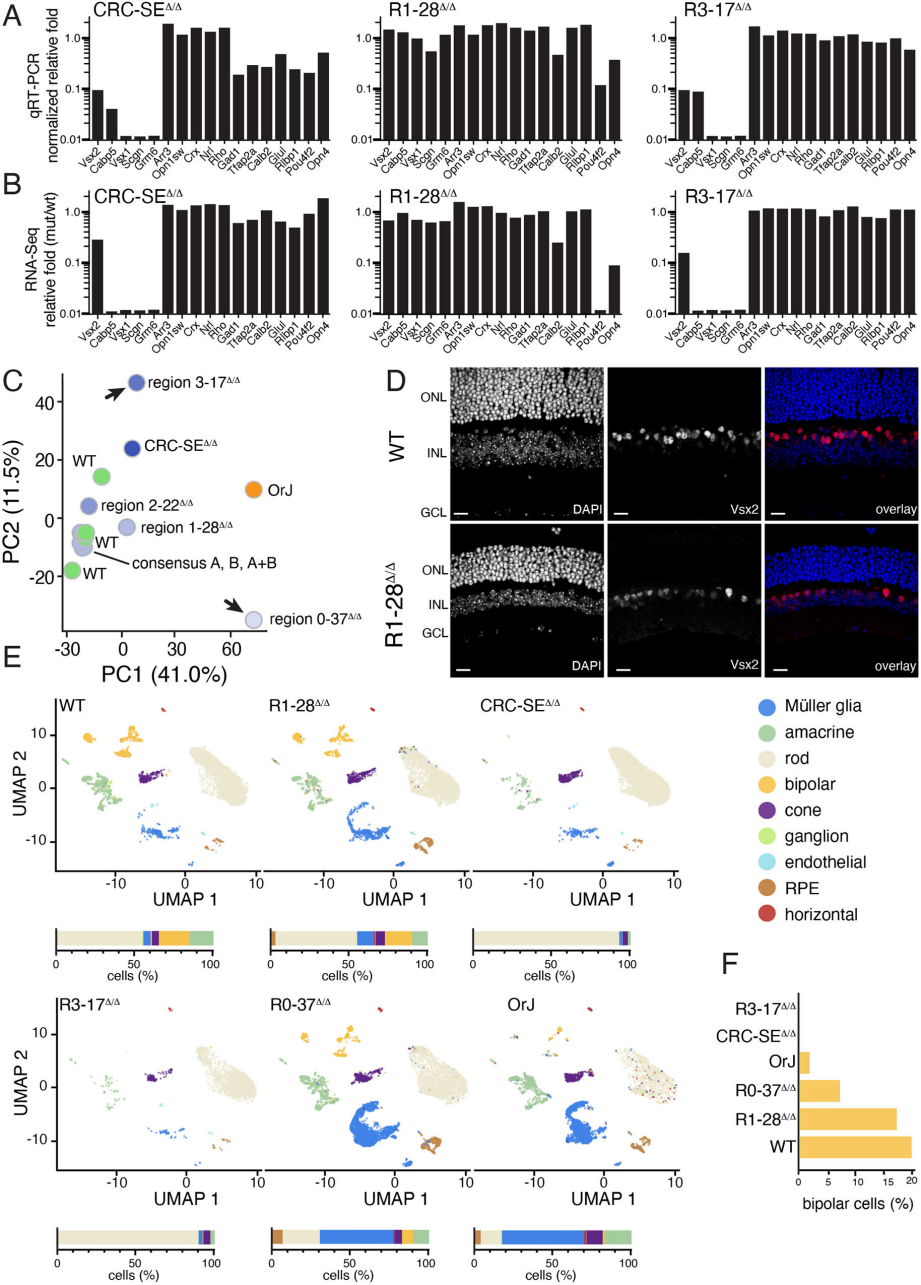
Separation of functional regions of the Vsx2 SE. **A**, **B** qRT-PCR and RNA-seq of genes for retinal cell types for each of the indicated strains. Bipolar cells are missing from the original SE deletion strain and the R3-17 strain while there is a defect in retinal ganglion cell formation in the R1-28 deletion. **C** Principal component analysis (PCA) of bulk RNA-seq for each deletion mouse strain. The three consensus binding mutants are stacked on top of each other and indistinguishable from WT. **D** Micrographs showing the expression of Vsx2 in bipolar cells and Müller glia in the adult wild type and R1-28 deletion strain. The retinal thickness is about half that of the wild type but most cell types are present. **E** UMAP plots for WT, R1-28, CRC-SE, R3-17, R0-37, and *orJ* retinae. Stack bar plots show the percentage of each cell type below each UMAP. **F** Bar plot of the percentage of bipolar neurons in each of the retinae shown in (**E**). Abbreviations: ONL, outer nuclear layer; INL, inner nuclear layer; GCL, ganglion cell layer; PC1, PC2, principal components 1 and 2. Scale bars: 25 μm.

To elucidate any changes in cell-type distribution across these eight deletion strains, we performed bulk RNA-seq, scRNA-seq, immunostaining and qRT-PCR for all retinal cell types in adult retina. R2-22 deletion and the three consensus binding site mutant strains had no discernable retinal defect even though ChIP-seq confirmed reduced binding in the double consensus mutant (Fig. 6A, Fig. S1, and Datasets S1–S3). R3-17 deletion fully recapitulated the loss of bipolar neurons in the original 32 kb Vsx2-SE deletion by RNA-seq, qRT-PCR, immunostaining, and morphologic analysis (Fig. 6A–C and Datasets S1–S3). Deletion of R1-28 led to a hypocellular retina that had no reduction in the proportion of EdU+ cells at E14.5, E17.5 or P0 and normal distribution of bipolar and amacrine neurons, cone and rod photoreceptors and Müller glia by RNA-seq, qRT-PCR, and immunostaining (Fig. 6A–E and Datasets S2–S4). There was a reduction in expression of some ganglion cell genes in the R1-28 deletion suggesting that there may be a defect in early cell cycle exit (Fig. 6B, C, E). However, bipolar cells and Müller glia were present in the R1-28 deletion strain (Fig. 6B, C, E, F). The R0-37 deletion mouse strain had microphthalmia but bipolar neurons and Müller glia were normal (Datasets S1–S3, S5). Therefore, there is a functional and physical separation of the region of the *Vsx2* SE that is required for retinal progenitor cell proliferation (R0-37 and R1-28) and for bipolar neuron formation (R3-17).

## Discussion

Over the past 2 decades, several of the major neurogenic transcription factors have been knocked out in mice resulting in dramatic defects in central nervous system development. However, a major challenge in the field is discerning the primary effect from secondary non-specific effects. For example, if neural progenitor proliferation is altered by knocking out a transcription factor in mice, there may be secondary non-specific effects on cell fate specification due to the evolutionarily conserved birth order of neurons and/or glia. Conditional knockout approaches can help discern between primary and secondary effects but it may be difficult to obtain *Cre* transgenic lines with the level of cell and/or developmental stage-specific control that is required. Our data presented here on the *Vsx2* super-enhancer provides an alternative strategy for elucidating the function of neurogenic transcription factors in development. A single super-enhancer contains all the modules required for expression of Vsx2 in retinal progenitor cells, Müller glia and bipolar neurons. By deleting those individual modules in mice using CRISPR-Cas9, we were able to specifically study the function of Vsx2 in the different cell types at distinct stages of development. Super-enhancers have been identified for many of the neurogenic transcription factors and using existing scATAC-seq and scRNA-seq along with enhancer profiling it is now possible to identify similar modules other super-enhancers that can be tested and validated in vivo.

### A modular super-enhancer

In our original study^35^, we did not delete the entire super-enhancer that was identified by H3K27Ac ChIP-seq because of the close proximity of the 5' end of the Vsx2 SE to the 3' end of the *Lin52* gene. Previous studies had shown that a region in that deletion contained a module that was important for bipolar expression using in vivo square wave electroporation^37,38^. Our analysis of R3-17 of the Vsx2 SE in genetically engineered mice in vivo agreed with their original experimental results and highlights the power of combining in vivo square wave electroporation with genetic engineering of mice using CRISPR/Cas9. The retinal progenitor cell regulatory elements were not identified in those prior studies and here we show that it resides adjacent to the *Lin52* gene but within the original Vsx2-SE defined computationally. Our in vivo studies allowed us to separate the bipolar gene expression regulatory module from the retinal progenitor cell module. This is in contrast to the Vsx2 *orJ* mutant mouse strain that has microphthalmia and lacks bipolar neurons. Previous analyses of SE elements in vivo have shown that multiple regions work cooperatively or in an additive manner to regulate gene expression^16,17,19,21^. Our identification of two modules (R0-37 and R1-28) important for retinal progenitor cell expression agree with the additive model and deletion of R0-37 through R1-28 led to a further reduction in eye/retina size. Previous studies suggest that there are proliferating retinal progenitor cells that do not express Vsx2^29,30^ so it is possible that any residual retinal progenitor cells in the R0-37/R1-28 deletion mouse retina would be those Vsx2– retinal progenitor cells.

### Bipolar neurons become rods and Müller glia in the Vsx2 SE deletion

Individual retinal progenitor cells can produce combinations of rods, bipolar neurons, and Müller glia^47–51^. Using complementary approaches, we showed that retinal progenitor cells produce more rods and Müller glia in the Vsx2 SE deletion mouse strain. The bipolar element has now been localized to R3-17 which overlaps with the region previously shown to control bipolar cell expression^37,38^. We cannot rule out the possibility that there are a small number of bipolar neurons born that undergo apoptosis in the Vsx2 SE deletion strain but if that is the case, it would be a minor contribution because we were not able to detect any significant increase in cell death in the Vsx2 SE deletion mouse strain.

### The core regulatory circuit of Vsx2

SEs and CRC-SEs are often identified from computational analyses of ChIP-seq data. However, a major debate in the field is focused on whether SEs and CRC-SEs are simply large contiguous arrays of cis-regulatory elements or entirely distinct entities. In either model, it is important to elucidate the function of the individual cis-regulatory elements in vivo especially for neurogenic transcription factors with complex temporal and spatial patterns of expression. The *Vsx2* CRC-SE is an important case in point. It meets the computational criteria for a CRC-SE^10^ because there are two Vsx2 consensus binding sites in R1-28 as well as other sites in the *Vsx2* CRC-SE. We showed by ChIP-seq that Vsx2 binds the R1-28 sites and mutating the sites in mice in vivo led to reduced Vsx2 binding. However, there was no discernable phenotype in the retina from mice lacking those sites. It is possible that the Vsx2 SE is a CRC-SE because the ChIP-seq showed other Vsx2 binding sites that overlapped with Isl1 and Prox1. It is possible that these transcription factors work together at R3-17 to regulate bipolar cell expression of Vsx2. Interestingly, the consensus binding sites for Vsx2 in R1-28 were sufficient to promote expression of a heterologous reporter gene in Müller glia even though there was no defect in Müller glial cell fate specification in the double consensus knockout. Thus, the Vsx2 binding sites in R1-28 appear to be sufficient for expression but not necessary.

### A systematic approach to dissection transcription factor SEs in neurogenesis

Our original deletion of the Vsx2 SE was imprecise because it was based only on identification of the super-enhancer domain and proximity to adjacent genes. In this study, we combined those previous ChIP-seq results with scRNA-seq and scATAC-seq to refine the developmental stage—and cell type–specific regions of the Vsx2 SE. Using this integrated approach, we were able to identify each module within the Vsx2 SE and discern their developmental stage—and cell type—specific activity in vivo. We propose that this approach using bulk RNA-seq and bulk ChIP-seq combined with scRNA-seq and scATAC-seq can be used to produce an atlas of multi-functional super-enhancers for neurogenic transcription factors during development. This is important because it would allow researchers to discern specific functions of transcription factors in particular cell types or at particular stages of development. Moreover, by comparing those essential domains across species, one can begin to elucidate the role of such super-enhancer sub regions in evolution of brain expression patterns and brain development. This provides an advantage to traditional gene knockout or conditional knockout approaches because it can be difficult to discern primary from secondary effects. With advances in human neural organoid development from embryonic stem cells, it is now possible to perform cross-species comparisons in mice and humans.

## Methods

### Mouse strains

All animal procedures and protocols were approved by the St. Jude Laboratory Animal Care and Use Committee under protocol number 393-100500. All studies conform to federal and local regulatory standards. Mice were housed on ventilated racks on a standard 12 h light–dark cycle. Wild-type C57BL/6 J mice were purchased from the Jackson Laboratory (Bar Harbor, ME, #000664). For timed pregnancy, individual male mice were housed with three females in a single cage. Plugged/pregnant females (identified by visual examination), were isolated and embryos or pups were harvested at the appropriate time. Both males and females were combined for this study. Conserved regions within the *Vsx2* SE were identified by examination of evolutionary conservation in the UCSC mm9 genome build. Vsx2 modified mouse models were created using CRISPR-Cas9 technology (Supplemental Information).

### Imaging

Images were taken with the Zeiss LSM 700 confocal microscope using the 40X lens. Brightness and contrast were modified for images presented in the figures for the IF studies. Raw original data are available for all datasets and probes.

### RNA isolation

RNA was extracted from individual TRIzol (Life Technologies) preparations via a phenol-chloroform extraction. Samples were first dissociated by pipetting retina and TRIzol vigorously. A 1:4 volume of chloroform (Sigma) was then added to each sample and incubated at room temperature for 3 min followed centrifugation at 12,000 3 g at 4 C for 15 min. The aqueous layer was then transferred to a siliconized Eppendorf tube followed by the addition of 2.0 mL glycogen (Roche) and 500 mL isopropanol (Fisher Scientific). Samples were incubated at room temperature for 10 min followed by centrifugation at 12,000 3 g at 4 C for 15 min. Samples were then washed twice with ice-cold 80% EtOH (Fisher) to remove salts, resuspended in DEPC H2O, and the concentration was determined with a NanoDrop (Thermoscientific).

Libraries were prepared from 500 ng total RNA with the TruSeq Stranded Total RNA Library Prep Kit according to the manufacturer’s directions (Illumina). Paired-end 100-cycle sequencing was performed on HiSeq 2000 or HiSeq 2500 sequencers according to the manufacturer’s directions (Illumina).

### qRT-PCR

cDNA was made from 200 ng of RNA from Vsx2SE-Consensus, Vsx2SE-DoubleConsensus, Vsx2SE-Region1, Vsx2SE-Region2, Vsx2SE-Region3, and Vsx2SE knockout retinae, as well as for their wild type and heterozygous littermates (Applied Biosystems 4387406). cDNA was loaded onto a Custom TaqMan Array Card (Applied Biosystems 4342249) run on a QuantStudio 7 Flex (ThermoScientific) system. “Undetermined” values were set to a Ct of 40 as the limit of detection of the assay.

### RNA-seq

RNA was quantified using the Quant-iT RiboGreen assay (Life Technologies) and quality checked by 2100 Bioanalyzer RNA 6000 Nano assay (Agilent,) 4200 TapeStation High Sensitivity RNA ScreenTape assay (Agilent,) or LabChip RNA Pico Sensitivity assay (PerkinElmer) prior to library generation. Libraries were prepared from total RNA with the TruSeq Stranded Total RNA Library Prep Kit according to the manufacturer’s instructions (Illumina, PN 20020599). Libraries were analyzed for insert size distribution on a 2100 BioAnalyzer High Sensitivity kit (Agilent Technologies,) 4200 TapeStation D1000 ScreenTape assay (Agilent Technologies,) or Caliper LabChip GX DNA High Sensitivity Reagent Kit (PerkinElmer.) Libraries were quantified using the Quant-iT PicoGreen ds DNA assay (Life Technologies) or low pass sequencing with a MiSeq Nano kit (Illumina) Paired-end 100-cycle sequencing was performed on a NovaSeq 6000 (Illumina). For PCA analysis, only protein-coding genes with the annotation level (https://www.gencodegenes.org/pages/data_format.html) in 1 (verified loci) and 2 (manually annotated loci) were included in the analysis. With input of read counts for all samples, counts per million mapped reads (CPMs) were obtained by using the function *cpm* in the edgeR package. Genes were removed when the corresponding CPMs for all samples were smaller than the CPM whose corresponding raw read count is 10. Then, the top 3000 most variable genes were selected by ranking the mean absolute deviation (MAD) of the log_2_-transformed CPMs in descending order. Based on these most variable genes, the PCA analysis was performed on the log_2_-transformed CPMs by using the *prcomp* function available in the standard R language. The top two principal components (PCs) were used to draw the PCA figures.

### Statistics and reproducibility

Littermates of both sexes were randomly selected for analyses. Investigators were blinded when scoring images. No statistical method was used to predetermine sample size. There were no instances in which repeat experiments yielded conflicting results, suggesting reproducibility of our experiments. GraphPad Prism 8 software was used to calculate statistical measures. No data were excluded from the analyses.

### Reporting summary

Further information on research design is available in the Nature Research Reporting Summary linked to this article.

## Supplementary information

Supplementary Information

Description of Additional Supplementary Files

Dataset 1

Dataset 2

Dataset 3

Dataset 4

Dataset 5

Reporting Summary

## Data Availability

The sequencing data generated in this study are publicly available in the GEO database under accession code “GSE169262”. The reference genome is mm10 and can be accessed by downloading the mouse Gencode release M22 at the following “FTP link [ftp://ftp.ebi.ac.uk/pub/databases/gencode/Gencode_mouse/release_M22/]”. All other relevant data supporting the key findings of this study are available within the article and its Supplementary Information files or from the corresponding author upon reasonable request. Source data are provided with this paper.

## Source data

Source Data

## Code availability

Code used to process all scRNA-seq data can be found on “Github [https://github.com/CodyRamirez/StJude_Scripts/tree/main/Vsx2_SE]” (10.5281/zenodo.5777750) under the MIT license.

## References

[1] Livesey FJ, Cepko CL (2001). Vertebrate neural cell-fate determination: lessons from the retina. Nat. Rev. Neurosci..

[2] Amano T (2009). Chromosomal dynamics at the Shh locus: limb bud-specific differential regulation of competence and active transcription. Dev. Cell.

[3] Kleinjan DA, van Heyningen V (2005). Long-range control of gene expression: emerging mechanisms and disruption in disease. Am. J. Hum. Genet..

[4] Mifsud B (2015). Mapping long-range promoter contacts in human cells with high-resolution capture Hi-C. Nat. Genet..

[5] Sanyal A, Lajoie BR, Jain G, Dekker J (2012). The long-range interaction landscape of gene promoters. Nature.

[6] Li Y (2014). CRISPR reveals a distal super-enhancer required for Sox2 expression in mouse embryonic stem cells. PLoS One.

[7] Zhou HY (2014). A Sox2 distal enhancer cluster regulates embryonic stem cell differentiation potential. Genes Dev..

[8] Visel A (2009). ChIP-seq accurately predicts tissue-specific activity of enhancers. Nature.

[9] Shen Y (2012). A map of the cis-regulatory sequences in the mouse genome. Nature.

[10] Aldiri I (2017). The dynamic epigenetic landscape of the retina during development, reprogramming, and tumorigenesis. Neuron.

[11] Pott S, Lieb JD (2015). What are super-enhancers?. Nat. Genet..

[12] Hnisz D (2013). Super-enhancers in the control of cell identity and disease. Cell.

[13] Loven J (2013). Selective inhibition of tumor oncogenes by disruption of super-enhancers. Cell.

[14] Whyte WA (2013). Master transcription factors and mediator establish super-enhancers at key cell identity genes. Cell.

[15] Blobel, G. A., Higgs, D. R., Mitchell, J. A., Notani, D. & Young, R. A. Testing the super-enhancer concept. *Nat. Rev. Genet.*10.1038/s41576-021-00398-w (2021). 10.1038/s41576-021-00398-w 34480110

[16] Hay D (2016). Genetic dissection of the α-globin super-enhancer in vivo. Nat. Genet..

[17] Hnisz D (2015). Convergence of developmental and oncogenic signaling pathways at transcriptional super-enhancers. Mol. Cell.

[18] Huang J (2018). Dissecting super-enhancer hierarchy based on chromatin interactions. Nat. Commun..

[19] Huang J (2016). Dynamic control of enhancer repertoires drives lineage and stage-specific transcription during hematopoiesis. Dev. Cell..

[20] Parker SC (2013). Chromatin stretch enhancer states drive cell-specific gene regulation and harbor human disease risk variants. Proc. Natl. Acad. Sci. USA.

[21] Shin HY (2016). Hierarchy within the mammary STAT5-driven Wap super-enhancer. Nat. Genet..

[22] Haubst N (2004). Molecular dissection of Pax6 function: the specific roles of the paired domain and homeodomain in brain development. Development.

[23] Castro DS (2011). A novel function of the proneural factor Ascl1 in progenitor proliferation identified by genome-wide characterization of its targets. Genes Dev..

[24] Nishida A (2003). Otx2 homeobox gene controls retinal photoreceptor cell fate and pineal gland development. Nat. Neurosci..

[25] Collinson JM, Quinn JC, Hill RE, West JD (2003). The roles of Pax6 in the cornea, retina, and olfactory epithelium of the developing mouse embryo. Dev. Biol..

[26] Marquardt T (2001). Pax6 is required for the multipotent state of retinal progenitor cells. Cell.

[27] Liu IS (1994). Developmental expression of a novel murine homeobox gene (Chx10): evidence for roles in determination of the neuroretina and inner nuclear layer. Neuron.

[28] Rowan S, Cepko CL (2004). Genetic analysis of the homeodomain transcription factor Chx10 in the retina using a novel multifunctional BAC transgenic mouse reporter. Dev. Biol..

[29] Vitorino M (2009). Vsx2 in the zebrafish retina: restricted lineages through derepression. Neural Dev..

[30] Buenaventura DF, Ghinia-Tegla MG, Emerson MM (2018). Fate-restricted retinal progenitor cells adopt a molecular profile and spatial position distinct from multipotent progenitor cells. Dev. Biol..

[31] Burmeister M (1996). Ocular retardation mouse caused by Chx10 homeobox null allele: impaired retinal progenitor proliferation and bipolar cell differentiation. Nat. Genet..

[32] Ferda Percin E (2000). Human microphthalmia associated with mutations in the retinal homeobox gene CHX10. Nat. Genet..

[33] Livne-Bar I (2006). Chx10 is required to block photoreceptor differentiation but is dispensable for progenitor proliferation in the postnatal retina. Proc. Natl. Acad. Sci. USA.

[34] Truslove GM (1962). A gene causing ocular retardation in the mouse. J. Embryol. Exp. Morphol..

[35] Norrie JL (2019). Nucleome dynamics during retinal development. Neuron.

[36] Saint-Andre V (2016). Models of human core transcriptional regulatory circuitries. Genome Res..

[37] Kim DS, Matsuda T, Cepko CL (2008). A core paired-type and POU homeodomain-containing transcription factor program drives retinal bipolar cell gene expression. J. Neurosci..

[38] Goodson, N. B., Kaufman, M. A., Park, K. U. & Brzezinski, J. A. T. Simultaneous deletion of Prdm1 and Vsx2 enhancers in the retina alters photoreceptor and bipolar cell fate specification, yet differs from deleting both genes. *Development*10.1242/dev.190272 (2020). 10.1242/dev.190272 PMC10666920 32541005

[39] Dhingra A (2008). Probing neurochemical structure and function of retinal ON bipolar cells with a transgenic mouse. J. Comp. Neurol..

[40] Hao Y (2021). Integrated analysis of multimodal single-cell data. Cell.

[41] Korsunsky I (2019). Fast, sensitive and accurate integration of single-cell data with Harmony. Nat. Methods.

[42] Welch JD (2019). Single-cell multi-omic integration compares and contrasts features of brain cell identity. Cell.

[43] Rowan S, Cepko CL (2005). A POU factor binding site upstream of the Chx10 homeobox gene is required for Chx10 expression in subsets of retinal progenitor cells and bipolar cells. Dev. Biol..

[44] Matsuda T, Cepko CL (2004). Electroporation and RNA interference in the rodent retina in vivo and in vitro. Proc. Natl. Acad. Sci. USA.

[45] Emerson MM, Cepko CL (2011). Identification of a retina-specific Otx2 enhancer element active in immature developing photoreceptors. Dev. biol..

[46] Jean-Charles N, Buenaventura DF, Emerson MM (2018). Identification and characterization of early photoreceptor cis-regulatory elements and their relation to Onecut1. Neural Dev..

[47] Wang S, Sengel C, Emerson MM, Cepko CL (2014). A gene regulatory network controls the binary fate decision of rod and bipolar cells in the vertebrate retina. Dev. Cell..

[48] Turner DL, Cepko CL (1987). A common progenitor for neurons and glia persists in rat retina late in development. Nature.

[49] Wang S, Cepko CL (2016). Photoreceptor fate determination in the vertebrate retina. Investig. Ophthalmol. Vis. Sci..

[50] Hafler BP (2012). Transcription factor Olig2 defines subpopulations of retinal progenitor cells biased toward specific cell fates. Proc. Natl. Acad. Sci. USA.

[51] Honnell, V. et al. Identification of a modular super-enhancer in murine retinal development. Github 10.5281/zenodo.5777750 (2021). 10.1038/s41467-021-27924-y PMC8752785 35017532

