# Supplementary material for: Identification of a modular super-enhancer in murine retinal development
Source: Nat Commun. 2022 Jan 11;13:253. doi: 10.1038/s41467-021-27924-y (PMC8752785; doi:10.1038/s41467-021-27924-y)
Supplement: Supplementary file 1 — Supplementary Information [file 41467_2021_27924_MOESM1_ESM.pdf]

Supplemental Figure 1

Figure S1. ChIP-seq for transcription factors found in bipolar neurons.

A) Drawing of the original Vsx2 CRC-SE and regions 0, 1, 2 and 3 (grey) in mm10. **B)** scATAC-seq peak tracks by cell type for adult and E14.5 retinae. In adult, there is a peak in region 0 for region Müller glia and a peak in region 3 for bipolar cells. In E14.5, there is a peak in region 0 for RPCs. **C)** ChIP-seq of adult retina for Isl1, Prox1, and two different antibodies for Vsx2. **D)** ChIP-seq of adult wt and double consensus mutant mice retinae. There is a reduction of binding in the double consensus retinae (arrows). **E)** Bar plot of mean with standard deviation of the percentage of PKCa immunopositive cells scored in retinal sections. The CRISPR was expressed from a plasmid with a bipolar-specific regulatory element and gRNAs were targeting R2-22 (negative control, unpaired two-sided t-test, p=0.0761) or R3-17 (unpaired two-sided t-test, p=0.0328) to eliminate the bipolar-specific element or the *Vsx2* coding region. N=3 biologically independent retina examined for each plasmid. **F,G)** Bar plot of mean with SEM of biological triplicate samples scored for EdU+, GFP+, Scarlet+/Scarlet+ cells.

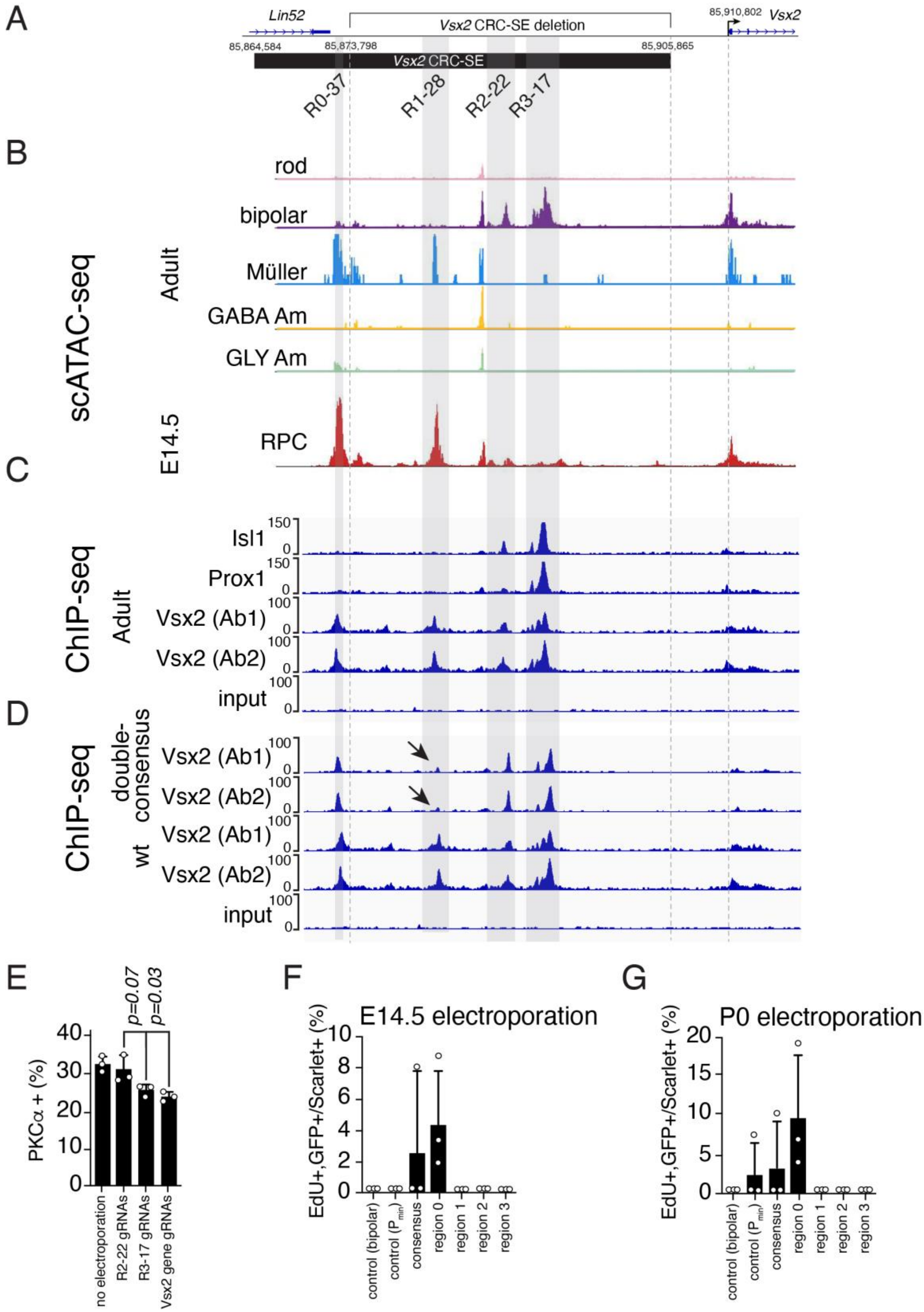

A
B
C
D
E
F
G
Lin52
Vsx2 CRC-SE deletion
85,910,802
Vsx2
85,864,584
85,873,798
85,905,865
Vsx2 CRC-SE
R0-37
R1-28
R2-22
R3-17
scATAC-seq
Adult
rod
bipolar
Müller
GABA Am
GLY Am
E14.5
RPC
ChIP-seq
Adult
Isl1
Prox1
Vsx2 (Ab1)
Vsx2 (Ab2)
input
ChIP-seq
double-consensus
wt
Vsx2 (Ab1)
Vsx2 (Ab2)
Vsx2 (Ab1)
Vsx2 (Ab2)
input
PKCα + (%)
p=0.07
p=0.03
no electroporation
R2-22 gRNAs
R3-17 gRNAs
Vsx2 gene gRNAs
E14.5 electroporation
EdU+,GFP+/Scarlet+ (%)
control (bipolar)
control (Pmin)
consensus
region 0
region 1
region 2
region 3
P0 electroporation
EdU+,GFP+/Scarlet+ (%)
control (bipolar)
control (Pmin)
consensus
region 0
region 1
region 2
region 3

Supplemental Figure 2

Figure S2. Gating strategy for flow cytometry.

A) DAPI negative cells were selected as live cells. **B)** Live cells were analyzed for GFP. **C)** Population statistics for gating. Percent GFP+ cells for each sample is in the Source Data file.

A B

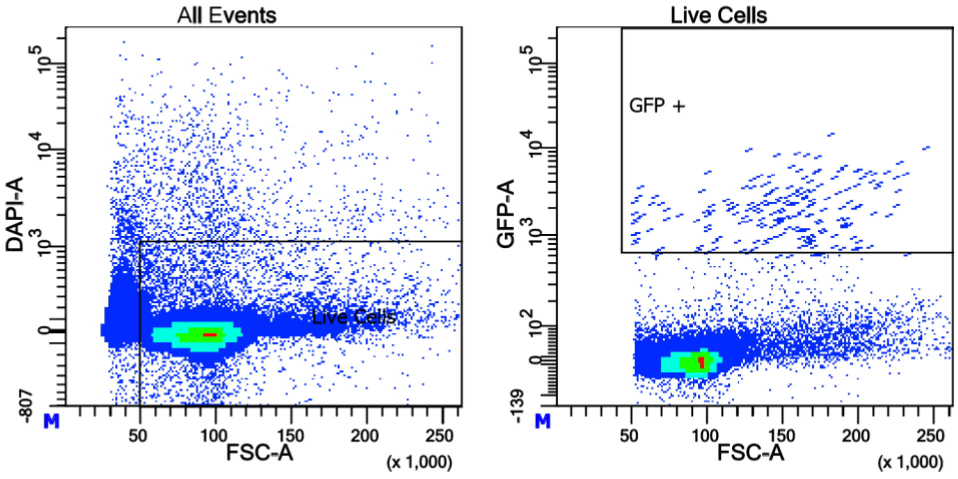

C

BD FACSDiva 8.0.1

Tube: _001

Population	#Events	%Parent	%Total
All Events	25,060	####	100.0
Live Cells	20,614	82.3	82.3
GFP +	193	0.9	0.8

Supplemental Methods

Generation of Vsx2-SE CRISPR gene-edited mice

Conserved regions within the Vsx2-SE were identified by examination of evolutionary conservation in the UCSC mm9 genome build. Vsx2 modified mouse models were created using CRISPR-Cas9 technology. Briefly, chemically modified sgRNAs (Synthego) were tested prior to embryo injection for activity in mouse Neuro-2a cells (ATCC, Cat. # CCL-131) stably expressing Cas9 and assayed by targeted next generation sequencing (NGS) as previously described[1]. Resulting NGS data was analyzed using CRIS.py (https://www.ncbi.nlm.nih.gov/pmc/articles/PMC6414496/). For animal model generation, ten 3-4-week-old C57BL/6J female mice from Jackson Labs were superovulated with 5 units of gonadotrophin from pregnant mare's serum (PMSG from ProSpec) and 48 hours later, with 5 units of human chorionic gonadotrophin (hCG from Sigma). After overnight mating with C57BL/6J males, the females were euthanized, and oocytes were harvested from the ampullae. The protective cumulus cells were removed using hyaluronidase, and the oocytes were washed and graded for fertilization by observing the presence of two pronuclei. A mixture of the respective sgRNAs, Cas9, and ssODNs consisting of either 50-75ng/ul of Cas9 mRNA (Trilink), 25-50ng/ul of sgRNA, and 5-10ng/ul of ssODN (IDT) or 30-60ng/ul of Cas9 Protein (St. Jude Protein Production Core), 10-20ng/ul of each sgRNA, and 5-10ng/ul of ssODN were injected into the cytoplasm or pronucleus of oocytes, respectively. The injected oocytes were then returned to culture media (M16 from Millipore or A-KSOM from Millipore) and later the same day transferred to day 0.5 pseudopregnant fosters (7-10-week-old CD-1 females from Charles River Laboratories mated to vasectomized CD-1 males). Pups were born after 19 days gestation and sampled at day 7-10 for genotyping via targeted NGS. Animals positive for the respective deletions were weaned at day 21 and at 6 weeks of age, they were backcrossed to C57BL/6J mice. Editing construct sequences and relevant primers are listed in Supplemental Table 1.

Table S1. gRNA sequences for generation of gene-edited mice.

Name	Sequence (5' to 3')
Vsx2-SE Region0 (R0-37)	
CAGE662.Vsx2.g25 sgRNA spacer	GUUAGACCUAGUCAGAACUC
CAGE663.Vsx2.g26 sgRNA spacer	GCAGGCCAUGUGCUCGUCGA
CAGE662.g25.663.g26.del *phosphothioate linkages	T*T*CTATCCTCCAAGACACCTAGGATTCCAGCA GCTCGTTGAATGGGCAAGCAAAGTCTCCCAAGT TCAGCCCTGAGTTCTGACAGAGCCTGAGTGAGA GTCTACCCTCGACGAGCACATGGCCTGCCAGAG GCTCCATTCCTCAGCTCGCTTA*C*G
CAGE662.DS.F	AAGATGGACTGCGGGTTGGACGGGG
CAGE663.DS.R	AGGCTACGTGTGTGTGTGTGTGTGT
Vsx2-SE Region1 (R1-28)	
JP60.VSX2.g4 sgRNA spacer	AGCUGGAGUACCUACAUUGU
JP61.VSX2.g7 sgRNA spacer	GUCUAGCUUGUGGAUUUCUU

JP60.g4.JP61.g7.sense.ssODN *phosphothioate linkages	T*G*TAATACCTGTAGTGGCCAGAAGAGGGCATCAGATCCCCAGGAGCTGGAGTACCTACATCTTTGGTTGGTGGTTCAGTCTCTGAGAGCCCCAGGGGCCCAGGTTTGTTGACTCTGTT*G*G
JP60.DS.F	TGTCTCCCCCTCCCTCCATCCCTCC
JP61.DS.R	GGGGACTGTGGAGCCTTTGAAGCCA
Vsx2-SE Region 0 + 1 (R0-37-R1-28)	
CAGE662.Vsx2.g25 sgRNA spacer	GUUAGACCUAGUCAGAACUC
JP61.VSX2.g7 sgRNA spacer	GUCUAGCUUGUGGAUUUCUU
CAGE662.Vsx2.F	AAGATGGACTGCGGGTTGGACGGGG
JP61.Vsx2.R	GGGGACTGTGGAGCCTTTGAAGCCA
Vsx2-SE ConsensusB	
SP255.VSX2.g7 sgRNA spacer	CCUGCCGAUAAUGCUUAAUU
SP255.F	GGGTTGGAAGAGCTGGGGAGGGGAG
SP255.R	GGTCCTTGGTGCCTCTTAGCTGTCCG
Vsx2-SE DoubleConsensus	
CAGE189.VSX2.g1 sgRNA spacer	ACAGCUAAUUAGAAGCCUAC
SP255.VSX2.g7 sgRNA spacer	CCUGCCGAUAAUGCUUAAUU
CAGE189.g1_255.g7_anti.ssODN *phosphothioate linkages	C*C*CACACAATGGTCTGCTTTGTGCCCACCTGCCGATAATGCTTAGGCGAGGAGTTGATTAATTTGAGGTAATTGACAGCTAGAAGCCTACAGGCTCATTAACTTGACATCTTGTCTTTGTGAATT*G*G
CAGE189.F	CCGGATCTGCCCAGCCCTCCCC
CAGE189.R	AGGGAGGCTGTTGCGGTGCACAG
Vsx2-SE Region2 (R2-22)	
BH33.VSX2.g51 sgRNA spacer	UGGUGAUGGAGCGCAUAGCG
BH34.VSX2.g21 sgRNA spacer	GGGCAGUGCAUCCCCUGUGG
BH33.g51.BH34.g21.sense.ssODN *phosphothioate linkages	C*C*AGCCACTGTCCCAGCCCGGGCTCTAAGTCTGACCCAAGCCGAAGTGCCTGCGCCCCGCTGGTGGGTGGGTGTGGACATCTTCCAGGCCCCAGGACACACCAGGAATCAAGAGGGAG*G*T
BH33.DS.F	GACGGTCTCCTGATGGATGTCTGCA
BH34.DS.R	CTCTCTCCAGCCCCCGGTGTCTGG
Vsx2-SE Region3 (R3-17)	
SNP300.VSX2.g5 sgRNA spacer	AAGAUGGGUGCAUGGUGCGG
SNP301.VSX2.g39 sgRNA spacer	AGACUACAUCUAGACGUGCU
SNP300.g5.SM301.g39.sense.ssODN *phosphothioate linkages	G*C*CCATCACTCTGTCCATCGGTCTGACTAAACAGTTTTGAATAAAGATGGGTGCATGGTGGCTAGGGCCTTCACGAGAACCTCAGCTGTGCGGCACCCTTGAGCCATTTGAACTCTCT*C*A
SNP300.DS.F2	CAAGTCAGAGTTGTTCAGGACG
SNP301.DS.R	TCCAGGGGGTACACGGAGAAACCCT

Vsx2-SE ConsensusB mice were generated using Vsx2-SE Double Consensus guides that only produced and indel in the ConsensusB site. Next, the injected oocytes were returned to culture media (M16 from Millipore or A-KSOM from Millipore) and later the same day transferred to day 0.5 pseudopregnant fosters (7-10 week old CD-1 females from Charles River Laboratories mated to vasectomized CD-1 males). Pups were born after 19 days gestation, then sampled at day 7-10 for genotyping. Animals positive for the respective deletions were weaned at day 21 and at 6 weeks of age, they were backcrossed to C57BL/6J mice. The primers used for genotyping are shown with Mi-Seq adaptors (underlined):

Forward

Vsx2SE-Consensus and Vsx2SE-Double Consensus
5'-TCGTCGGCAGCGTCAGATGTGTATAAGAGACAGGGGTTGGAAGAGCTGGGGAGG
GGAG-3'

Vsx2SE-Region0
5’-TCGTCGGCAGCGTCAGATGTGTATAAGAGACAGAAGATGGACTGCGGGTTGGAC
GGGG-3’

Vsx2SE-Region0+1
5’-TCGTCGGCAGCGTCAGATGTGTATAAGAGACAGAAGATGGACTGCGGGTTGGAC
GGGG-3’

Vsx2SE-Region1
5'-TCGTCGGCAGCGTCAGATGTGTATAAGAGACAGTGTCTCCCCCTCCCTCCATCCCT
CC-3'

Vsx2SE-Region2
5'-TCGTCGGCAGCGTCAGATGTGTATAAGAGACAGGACGGTCTCCTGATGGATGTCT
GCA-3'

Vsx2SE-Region3
5'-TCGTCGGCAGCGTCAGATGTGTATAAGAGACAGTCCATCCCAAGGACCCCACTGG
-3'

Vsx2SE
5'-ACAGGCTCTGACCTTCCTGGAAGCCCCGC-3'

Reverse:

Vsx2SE-Consensus and Vsx2SE-Double Consensus
5'-GTCTCGTGGGCTCGGAGATGTGTATAAGAGACAGGGTCCTTGGTGCCTCTTAGCT
GTCCG-3'

Vsx2SE-Region0
5'-GTCTCGTGGGCTCGGAGATGTGTATAAGAGACAGAGGCTACGTGTGTGTGTGTGTGTGT-3'

Vsx2SE-Region0+1
5'-GTCTCGTGGGCTCGGAGATGTGTATAAGAGACAGGGGGACTGTGGAGCCTTTGAAGCCA-3'

Vsx2SE-Region1
5'-GTCTCGTGGGCTCGGAGATGTGTATAAGAGACAGGGGGACTGTGGAGCCTTTGAAGCCA-3'

Vsx2SE-Region2
5'-GTCTCGTGGGCTCGGAGATGTGTATAAGAGACAGCTCTCTCCAGCCCCCGGTGTCTGG-3

Vsx2SE-Region3
5'-GTCTCGTGGGCTCGGAGATGTGTATAAGAGACAGTCCAGGGGGTACACGGAGAAACCCT-3'

Vsx2SE
5'-CTCAGGAGGTTACAAGGAGGTGTAG-3'

We also generated a set of primers internal to the deletion to serve as a wild-type primer set to distinguish heterozygous and homozygous deletion mice.

Forward WT:

Vsx2SE-Region0
5’-GCCACTTTGGAAGTGTGCC-3’

Vsx2SE-Region1
5’-CAAGATCTGACCCCCTCTTCTGGAG-3’

Vsx2SE-Region2
5’-GTAGGGAACAAGTGAGGTAATGGGAGG-3’

Vsx2SE-Region3
5’-CTTCTGTAAAGTGGGTGTTCGTCATAGC-3’

Vsx2SE
5'-CATAACTGGCTGTATTCTGTGTGACTC-3'

Reverse WT:

Vsx2SE-Region0

5’-GCATTGGGGTGTTCTTGTGC-3’

Vsx2SE-Region1
5’-GTGGTTAGAAGTACGTACTGCTCTTGTG-3’

Vsx2SE-Region2
5’-CTCCAAAGTTGAGGTCACAGACCTG-3’

Vsx2SE-Region3
5’-GAAGCAGACAGGCAAACAGAGACTAAG-3’
Vsx2SE
5'-CTTACATCCTTTGACCCTGGCTATG-3'

The deletion junctions for the mouse sublines are:

Double Consensus:
AGCCTGTAGGCTTC/TAGCTGTCAATTACCTCAAATTAATCAACTCCTCGCC/TAAGCA TTATCGGCAGGTGGGC

ConsensusA:
ATCAACTCCTCGC/TAAGCATTATCGGCAGG
ATCAACTCCTCGC/(T)TAAGCATTATCGGCAGG

ConsensusB:
AGCCTGTAGGCTTC/TAGCTGTCAATTACCT

Region0:
CTCCCAAGTTCAGCCC/CTGCCAGAGGCTCCAT
CAAGTTCAGCCCTGAG/CGACGAGCACATGGCC*
AAGTTCAGCCCTGAGT/GCACATGGCCTGCCAG

Region0+1:
TGAATGGGCAAGCAAA/TGACTCTGTTGGTCTT
CCCAAGTTCAGCCCTG/TTTGGTTGGTGGTTCA
TCTCCCAAGTTCAGCC/CTTTGGTTGGTGGTTC

Region1:
GGGCATCAGATCCCCAGGAGC/TGGTGGTTCAGT*
GGGCATCAGATCCCCAGGAGC/TGGTTGGTGGTTCAGT*
GGGCATCAGATCCCCAGGAGCTGGAGTACCTAC/CTTTGGTTGGTGGTTCAGT

Region2:
CCGAAGTGCC/TGGTGGGTGGGTG
CCGAAGTGCCTGCGCC/GGTGGGTGGGTG
Region3:

TTGAATAAAGATGGG/TGCTAGGGCCTTCACGA
TTGAATAAAGATGGGTGCATGGTG/TGCTAGGGCCTTCACGA*

Sublines annotated with an * were used for scRNA-seq as detailed below.

GFP Reporter Mice

Cell-type-specific GFP mice[2-5] were crossed with *Vsx2-SE*$^{\Delta/\Delta}$ mice. *Vsx2-SE*$^{+/\Delta}$;celltype-GFP mice were intercrossed to generate *Vsx2-SE*$^{+/+}$*;*celltype-GFP, *Vsx2-SE*$^{+/\Delta}$;celltype-GFP, *Vsx2-SE*$^{\Delta/\Delta}$*;*celltype-GFP pups.

Retina Dissociation

Retina dissociation buffer was prepared by adding 40 U papain (Worthington CAT#LS003119) to 400 uL of papain buffer and incubating at 37°C for 15 min. Retinae were individually dissected in retinal explant media (REM) and placed on ice. Four hundred microliters of buffer was added to each retina and incubated at 37°C. To dissociate the retina, tubes were agitated twice at 5 min intervals and 40 uL of DNAse solution (DS) was added and incubated at 37°C for an additional 5 min. The cell suspension was filtered through a 40-µm cell strainer (Falcon CAT#352340) and the filter was washed with PBS to bring the total volume to 1.4 mL.

Flow cytometry

Cells were evaluated on a FACS Aria Fusion (Becton Dickinson) flow cytometer. Cell death was assessed using a forward scatter versus DAPI plot (Figure S2). Data was analyzed using Diva software (Becton Dickinson). Two mice totaling 4 retina were used for each condition with the exception of Grm6-GFP in which 3 retina were used. Here, one retina was discarded prior to flow cytometry due to poor dissection of the tissue. Bar plot displays mean with SEM.

Caspase-3 Scoring

For each genotype of littermates, 3 images were collected for each of 2 sections per retina. The fields were selected randomly using the DAPI channel in order to minimize bias in the Caspase channel. Images were collected and then total nuclei and Caspase+ nuclei were scored. Nuclear fragments were not scored. The number of Caspase + nuclei across the 3 images on a given section were combined and the total number of nuclei were combined and the ratio and percentage were calculated. The data for the two sections were averaged and the standard deviation was calculated. The individual datapoints from independent sections were plotted along with mean and SD.

Immunostaining

Retinae from adult littermates were isolated and fixed in 4% PFA overnight at 4C. Retinae were washed 3x with PBS and then embedded in 4% LMP agarose in PBS for vibratome sectioning at 50 mm. They were incubated in block solution for 1 hr at room temperature and then in primary antibody in block solution overnight at 4C.

Antibody	Company	Catalog Number	Dilution
Calretinin	Chemicon International	MAB1568	1:100
Calbindin	Sigma	C9848	1:100
Pax6	DSHB	Pax6	1:500
PSD95	Affinity Bioreagents	MA1-046	1:100
GFAP	Sigma	G3893	1:100
Bassoon	Stressgen	VAM-PS003	1:500
HPC-1	Sigma	S0664	1:500
Rhodopsin	Custom-made	N/A	1:500
Brn3a	Santa Cruz	sc-8429	1:1000
PKC-a	Upstate	05-154	1:5000
GS	BD Pharmingen	610518	1:100
TH	Pel-Freez	P40101	1:500
Ribeye	Custom-made	N/A	1:100
Cone A	Millipore	AB15282	1:5000
Recoverin	MIllipore	AB5585	1:5000
Piccolo	Synaptic Systems	142002	1:100
GaO	Santa Cruz	sc-387	1:5000
Vsx2	Exalpha	X1180P	1:200
Vsx2	Exalpha	X1179P	1:200
PH3	Sigma	06-570	1:500
Sox2	MIllipore	AB5603	1:1000
Otx2	Santa Cruz	sc-30659	1:200
anti-GFP	Invitrogen	A21311	1:500
Caspase-3	BD Pharmingen	559565	1:1000
pH3	Sigma	06-570	1:500

Vibratome sections were washed twice in PBS and then incubated in secondary antibody for 1 hour at room temperature. All secondary antibodies were incubated a dilution of 1:500 in the appropriate block solution. We used donkey anti-mouse (Vector Labs BA2000), goat anti-rabbit (Vector Labs BA-1000), and rabbit anti-sheep (Vector Labs BA-6000). After secondary antibody, they were washed twice with PBS and incubated with ABC reagent (Vector Laboratories, Cat. # PK6100) for 30 minutes. We then used tyramide Cy3 (PerkinElmer, Cat. # FP1046) for 10 minutes at room temperature and washed 2x in PBS followed by DAPI at 1:1000

in PBS. Slices were mounted in Prolong gold reagent and imaged on a Zeiss LSM700 confocal microscope.

Retroviruses and retinal cultures

Retroviruses and retinal culture procedures are described elsewhere[6-8]. LIA-E encodes alkaline phosphatase, which is suited for in vivo studies of cell fate specification and differentiation, and NIN-E encodes nuclear lacZ and is ideal for analysis of proliferation of retinal progenitor cells.

LIA-E: There are 6 cell types and 9 samples with 3 genotypes. The genotype of *Vsx2-SE*$^{+/+}$, *Vsx2-SE*$^{+/\Delta}$, *Vsx2-SE*$^{\Delta/\Delta}$ covered 2, 5, and 2 samples (mice), respectively. The cell type prevalence of clones was tested using chi-squared test. P value was computed following permutations of all possible genotype arrangements across the 9 samples.
We find significant differences in cell type distribution according to genotype (p = 0.0053). Notably, bipolar cell + rod and > 2 rods were absent in *Vsx2 SE*$^{\Delta/\Delta}$ while they were present in the other two genotypes. The absence of bipolar cell + rod and > 2 rods was largely filled by an increased relative prevalence of Müller glia+rod in the *Vsx2-SE*$^{\Delta/\Delta}$ genotype.

NIN-E: We calculated total number of clones and median clone size for each sample across the 3 genotypes. There were 5 samples in each genotype. For the cell number of 10-20 and > 20, 15 and 25 were replaced, respectively. There are 15 samples (mice) with 12 cell levels in each sample. We conducted a Kruskall-Wallis test for total clones and median clone size among the genotypes. We do not find significant differences in number of clones (p = 0.784) or median number of cells per clone (p = 0.6634) according to genotype.

Developmental Time Course

Animals heterozygous for the intended deletion were crossed. Vsx2-SE and Region1 animals (P0, P3, P7) or their mothers (E14.5, E17.5) were injected with EdU (10 ug/g for 10 mM solution) (Invitrogen A10044) 1-hour prior to harvesting the retina. Retina were fixed by 4% PFA overnight, embedded by O.C.T. Compound (Scigen 4583), and cryosectioned at 10 um. Sections were fixed by 4% PFA, washed with 3% BSA-PBS, incubated with 0.5% Triton X-100 in PBS (Sigma T9284), and washed again. Cryosections of E14.5, E17.5 and P0 littermate retinae for EdU and pH3 were imaged using a Zeiss LSM 700 confocal microscope using a 40X lens. Two images from each mouse were scored by manually counting the proportion of EdU or pH3 cells with DAPI. Bar plots display the mean of manual scoring.

Retina dissociation and Scoring

P0, P3, or P7 *Vsx2*-SE and *Vsx2*-SE Region1 retinae were dissociated with Trypsin (Sigma-Aldrich, T9935), and then incubated with soybean-trypsin inhibitor (Sigma-Aldrich, T6522) and DNaseI (Sigma-Aldrich, D4513). After dissociation, explant culture medium (25 ml of FBS, 2.5 ml of HEPES, 2.5 ml of penicillin-streptomycin and 125 µl of insulin in 219.9 ml of DMEM/F12 with GlutaMAX) was added to the cell suspension to 0.6 mL. Cells were added to chamber slides, 95uL/well and incubated for 30 min at 37 C. They were then fixed by 4% PFA overnight at 4 C, washed by PBS-/-. EdU labeling was performed per manufacturer's instructions (Click-iT

EdU imaging kit, Invitrogen, catalog C10338), and DNA was stained with 0.2 µg/ml DAPI (Sigma-Aldrich). Images were taken with the Keyence microscope (BZ-X710) using a 20X lens. Two different areas of each chamber were scored blindly. 250 cells marked by DAPI were counted along with corresponding Cy3 staining. Bar plot displays the mean of retinae from littermates for dissociated cell scoring. At P0 n=8, P3 n=7, P7 n=7.

Scatterplot

A scatterplot was generated by comparing the log-transformed FPKMs of the knockout (KO) sample against those of the wildtype (WT) one. The R^2 value for the Pearson correlation coefficient was calculated by using the cor() function in R.

TF Binding Sites

We extracted sequence for deleted super-enhancer region (Mice genome Mm10, chr12:84532044-84564910) using fastaFromBed from BEDTools (version 2.24.0)[9]. Then we scanned motifs from TRANSFAC database[10] by FIMO --no-qvalue --thresh 1e-4) from MEME suite (version 4.11.3)[11].

Cloning Enhancers

Regions 1, 2, and 3 from mm9 build were inserted into pUC57 vectors with SalI/HindIII cloning sites (synthesized by GenScript). Enhancer candidates were chosen based on strong evolutionary conservation across vertebrates. Underlined sequences were added to the 5' and 3'.

Vsx2SE-Region0 sequence (length 983 bp):

GTCGACACTAGGTCTAACTGGCTGTTCATATCTGTTTAGACCTTTGCCACTTT…TGC
AGTGAGGTTCTTACCACCATGTCAAAGAGAATTTACCTCGAGAAGCTT

Vsx2SE-Region1 sequence (length 3135 bp):

GTCGACTAAATAAATAAATAAAAAAAAGAACCAAATTCACAAGAGCA…AGTACAT
ATATACACGCATGCCAGTACACATATACATGCATGCCAGCTCGAGAAGCTT

Vsx2SE-Region2 sequence (length 3368 bp):

GTCGACGAATCTTATAATAAAAGTCATTATTTTGTATAATTAATATATA…TAGAATT
CAAGGAATGGCTTTTTTTTAAAGATTTATTTATTTATTATATGTAAGCTCGAGAAGCT
T

Vsx2SE-Region3 sequence (length 4087 bp):

GTCGACCGGCTTTTTTTTTTTTTTTTTTTTTTTTTTTTGACAAAGTCTCATTGTATAC
…AGGCTTCCCCGTATGTCTGTAATCCCAGCTACTCTGACTCGAGAAGCTT

The Vsx2SE-DoubleConsensus sequence (298 bp) was isolated from wildtype gDNA using forward and reverse primers which also include a leader sequence and restriction site (underlined). TOPO TA Cloning (Invitrogen, pCR2.1-TOPO) was then used.
F: TAAGCAGTCGACGGGTTGGAAGAGCTGGGGAGGGGAG
R: TAAGCACTCGAGGGTCCTTGGTGCCTCTTAGCTGTCCG

The plasmids were transformed and CsCl prepped. Diagnostic digests and Sanger sequencing were used for plasmid validation.

The plasmids were cut using SalI (NEB R0138S) and XhoI (NEB R0146S) with Buffer 3.1 (NEB B7203S) and cloned into the SalI and XhoI sites of Chx10 (164bp crit reg+SV40 bas prom)-GFP-IRES-AP. Removal of the 164 bp critical region constituted the negative control plasmid. Chx10 (164bp crit reg+SV40 bas prom)-GFP-IRES-AP[12] constituted the positive control plasmid. Diagnostic digests and sequencing were used for plasmid validation.

Chx10 (164 bp crit reg+SV40 bas prom)-GFP-IRES-AP was a gift from Connie Cepko (Addgene plasmid # 18808 ; http://n2t.net/addgene:18808 ; RRID:Addgene_18808).

GFP Reporter Assay

0.5 uL of plasmid mixture (2 ug/uL of enhancer plasmid and 0.5 ug/uL of a pCig2-H3.3-scarlet plasmid, a normalization control generously gifted to us from the Solecki Lab, resuspended in HBSS (Corning, 21-022-CV)) was co-electroporated into the sub retinal space of C57/BL6 mice at P0. Mouse retinae were harvested at P21 for GFP amplification immunostaining. Experiments were performed in biological triplicates for each enhancer plasmid.

One 40X confocal image from three retina were scored for each construct. GFP+, Scarlet+ cell types were counted and divided by Scarlet+ (electroporation control) cells to calculate the percentage of GFP+ cells for each cell type. Cells were assigned to specific cell types based on their location and morphology. Bar plot displays mean and SD for each cell type for each construct.

Electroporation and scoring

Enhancer plasmids previously described above (Double Consensus, Region0, Region1, Region2, Region3), the control (bipolar)[12], and the control (P_{min}) were individually square wave electroporated with the H3.3 Scarlet electroporation control. Bar plot displays mean with standard deviation.

In vivo: 0.5 uL of the plasmid mixture (2 ug/uL of enhancer plasmid and 0.5 ug/uL of a pCig2-H3.3-scarlet plasmid resuspended in HBSS (Corning, 21-022-CV)) was injected into the subretinal space of P0 mouse pups and square-wave electroporated. 48 hours later the pups received an intraperitoneal injection of EdU (10 ug/g for 10 mM solution) (Invitrogen A10044). Retinae were harvested and fixed after 1 hour.

In vitro: E14.5 mouse retinae were harvested from timed breedings and square-wave electroporated in vitro (1 ug/uL of enhancer plasmid and 0.25 ug/uL of a pCig2-H3.3-scarlet plasmid) as previously described[13]. Retina were transferred to nuclepore tracketch membranes (Whatman) floating on 2 mL of retinal explant media. Retinal explant media contains DMEM/F12 (Gibco, 21041-025) supplemented with 10% FBS (Biowest, S1620), 5 ug/mL Insulin (Sigma, 11061-68-0), 1% Pen/Strep (Gibco, 10378-016), and 2% 1M HEPES (Lonza, 17-737E). Retinal explant cultures were maintained by adding 10 uL of retinal explant media to each retina at 24 hours. After 48 hours, EdU (1:1000 dilution of 10 mM solution in retinal explant media) (Invitrogen A10044) was added to the cultures. 1 hour later, retinae were fixed.

Retinae were fixed by 4% PFA for 1 hour at 4°C, embedded by O.C.T. Compound (Scigen 4583), and cryosectioned at 10 um. Sections were fixed by 4% PFA, washed with 3% BSA-PBS, incubated with 0.5% Triton X-100 in PBS (Sigma T9284), and washed again. EdU labeling was performed per manufacturer's instructions (Click-iT EdU Alexa Fluor 647 imaging kit, Invitrogen, catalog C10340) and sections were stained with DAPI (Sigma-Aldrich). Cryosections were imaged using a Zeiss LSM 700 confocal microscope using a 20X lens. One confocal image from three biological replicates were scored for each plasmid by manually counting the cells positive for both EdU, GFP, and Scarlet and dividing by the total number of Scarlet positive cells. Bar plot displays mean with SEM. N=3 biological replicates for each construct at E14.5 and P0 timepoints.

Cas9 Electroporation and scoring

Glycerol stock of mammalian single-gRNA expression vectors and mammalian Cas9 expression vectors (synthesized by Vector Builder) were thawed and spread on ampicillin agar plates. One colony from each vector was picked, CsCl prepped, validated by Sanger sequencing, and 0.5 uL of plasmid mixture was electroporated into the subretinal space of P0 mouse pups.

Cas9 Vector:
164+SV40 sequence-
TTCGAGAAGAGCACTGGCTGGGGCTGCTTGCCCGCTAATCCCAGCTGCCATTAAAAT
ATTAAAGATAAATCTAATCGTCTCTTTATCCAAAATAAGCGACTTTTGTGTGGGGAG
AAAACGTCTAACCCCTTAGGAGGAGAATTAGTTCTAATGCATCAAATGGAATTGCTC
GAGATCTGCGATCTGCATCTCAATTAGTCAGCAACCATAGTCCCGCCCCTAACTCCG
CCCATCCCGCCCCTAACTCCGCCCAGTTCCGCCCATTCTCCGCCCCATCGCTGACTAA
TTTTTTTTATTTATGCAGAGGCCGAGGCCGCCTCGGCCTCTGAGCTATTCCAGAAGTA
GTGAGGAGGCTTTTTTGGAGGCCTAGGCTTTTGCAAA

Region2:
40 ug pRP[Exp]-{164+SV40}>hCas9: 120 ug pRP[gRNA]-U6>{R2Forward}: 120 ug pRP[gRNA]-U6>{R2Reverse}: 20 ug pCig2-H3.3-Scarlet

R2Forward gRNA sequence- TGGTGATGGAGCGCATAGCG

R2Forward gRNA sequence- GGGCAGTGCATCCCCTGTGG

Vsx2 Coding:
40 ug pRP[Exp]-{164+SV40}>hCas9: 80 ug pRP[gRNA]-U6>{VC1}: 80 ug pRP[gRNA]-U6>{VC2}: 80 ug pRP[gRNA]-U6>{VC3}: 20 ug pCig2-H3.3-Scarlet

VC1 gRNA sequence- TGAGTCGGGAAGGAAGCCCG

VC2 gRNA sequence- CTGGAGAAGAGCAGTTCCG

VC3 gRNA sequence- GTCTGTTTCTAGAGTCGTG

Vsx2 Enhancer:
40 ug pRP[Exp]-{164+SV40}>hCas9: 80 ug pRP[gRNA]-U6>{VE1}: 80 ug pRP[gRNA]-U6>{VE2}: 80 ug pRP[gRNA]-U6>{VE3}: 20 ug pCig2-H3.3-Scarlet

VE1 gRNA sequence- CTATGTGGTAAGACCATGG

VE2 gRNA sequence- AAACAGAAAGTGGAGTACGG

VE3 gRNA sequence- AAAACGTCTAACCCCTTAGG

gRNAs targeting VE (Vsx2 Enhancer) and VC (Vsx2 Coding) were designed by the J. A. Brzezinski laboratory[14].

Retina were harvested, fixed in 4% PFA for 1 hour, vibratome sectioned, and immunostained using the PKCa antibody as described above (Flourescein Tyramide, Perkin Elmer CAT#FP1018). Sections were imaged with a Zeiss LSM 700 confocal microscope using a 40X lens. Three confocal images were scored from three biological replicates for each plasmid preparation. The proportion of bipolar cells present was quantified by counting the number of PKCa positive cells and dividing by the number of DAPI positive cells in the INL of Scarlet positive retina, the electroporation control. Three representative images from adjacent regions of unelectroporated retina indicated by the absence of Scarlet were scored as "no electroporation." Bar plot displays mean and SD.

Single cell RNA-seq and ATAC-seq

ScRNA-seq sample information

In total, 14 mice were used for this analysis (see table below). All mice have the C57BL/6J background except for the 129S1/Sv-*Vsx2*$^{or\text{-}J}$/J mouse strain from JAX stock #000395 [15] (referred to as *orJ*). Biological replicates were included for wildtype, *Vsx2 SE*$^{\Delta/\Delta}$, *Vsx2SE Region1*$^{\Delta/\Delta}$, and *Vsx2SE Region0*$^{\Delta/\Delta}$. One of the *Vsx2SE Region3*$^{\Delta/\Delta}$ replicates failed quality control and so that sample data is not included. Deletion mice used in this analysis are from the same subline with an exception of one *Vsx2SE Region1*$^{\Delta/\Delta}$ sample which differs from the subline consistently used throughout the manuscript by 4 additional base pairs on the 5' end (see deletion mouse junctions in Supplemental Information above, scRNA-seq sublines annotated with an *).

E14.5 retina	*Vsx2-SE*$^{+/+}$	
	Vsx2-SE$^{\Delta/\Delta}$	
Adult rod depleted retina	*Vsx2-SE*$^{+/+}$;Nrl-GFP	Littermates
	Vsx2-SE$^{\Delta/\Delta}$*;*Nrl-GFP	
Adult region deletion retina	*Vsx2-SE Region1*$^{+/+}$	Littermates
	Vsx2-SE Region1$^{\Delta/\Delta}$	
	Vsx2-SE Region1$^{\Delta/\Delta}$	
	Vsx2-SE$^{\Delta/\Delta}$	Littermates
	Vsx2-SE$^{\Delta/\Delta}$	
	Vsx2-SE Region3$^{\Delta/\Delta}$	
	Vsx2-SE Region0$^{+/+}$	Littermates
	Vsx2-SE Region0$^{\Delta/\Delta}$	
	Vsx2-SE Region0$^{\Delta/\Delta}$	
	orJ	

Retinae were dissected and each pair of retina was dissociated with 40U papain (Worthington CAT#LS003119) in 400uL of papain buffer (1mM L-cysteine with 0.5mM EDTA in PBS /) at 37C for 7 minutes with aggitation every 5 minutes. 40 mL of Dnase was added and the cells were incubated for an additional 5 min in at 37C. *Vsx2SE Region1*$^{+/+}$*, Vsx2SE Region1*$^{\Delta/\Delta}$, and E14.5 *Vsx2-CRC SE*$^{\Delta/\Delta}$ samples were filtered through a 40-mm mesh cell strainer and added to a 5 mL of BSA cushion medium (4% BSA in Retinal Explant media) and spun at 500g for 10 min at 4C to clear any debris. The supernatant was aspirated. All samples were resuspended in 400uL of retinal explant media and the concentration was determined by hemocytometer. GFP+ cells from *Vsx2-SE*$^{+/+}$;Nrl-GFP and *Vsx2-SE*$^{\Delta/\Delta}$;Nrl-GFP samples were sorted out of the samples by running them on the microfluidic chip cell sorter (On-chip Sort) twice. We first gated for size, removing debris, then sorted all cells that were GFP- for scRNA-seq.

Approximately 10,000 cells from each sample were taken and loaded onto the 10x chromium controller for single cell RNA sequencing analysis which was completed according to the 10x genomics protocol.

scRNA-seq data processing

Sequences from each individual Illumina sequencing dataset were demultiplexed using bcl2fastq v2.20.0.422 (Illumina). Sequencing reads were processed using 10X Genomics Cell Ranger version 6.0.0[16], with reads mapping to the mouse reference genome mm10 version3.0.0 (10x Genomics). Quality control (QC) filtering, clustering, dimensionality reduction, visualization, and differential gene expression were performed using Seurat v4.0.4[17] with R v4.1.0. Each dataset was filtered so that genes that were expressed in at least three or more cells were included in the final dataset. Cells were excluded if they: (1) expressed greater than or equal to 25 percent of mitochondrial genes, (2) had fewer than 200 genes expressed (presumed to be droplets or

cellular debris) or (3) expressed greater than or equal to 6,000 genes (for all adult mouse wild-type retina datasets) or 10,000 genes (for all E14.5, *Region0*$^{\Delta/\Delta}$, and *orJ* datasets). Datasets were individually log-normalized using Seurat's NormalizeData with default parameters. Cell cycle scoring was conducted using the associated S and G2M phase gene list from Tirosh et al 2015 using the CellCycleScoring command in Seurat. We calculated 3,000 features that exhibit high cell-to-cell variation in the dataset using Seurat's FindVariableFeature function. Next, we scaled the data by linear regression against the number of reads using Seurat's ScaleData function with default parameters. The variable genes were projected onto a low-dimensional subspace using principal component analysis using Seurat's RunPCA function with default parameters. The number of principal components (Npcs) were selected based on inspection of the plot of variance explained (Npcs = 30).

Datasets were integrated using Harmony with default parameters[18]. A shared-nearest-neighbor graph was constructed based on the Euclidean distance in the low-dimensional subspace using Seurat's FindNeighbors with dims = 1:30 and default parameters. Integrated datasets then underwent non-linear dimensional reduction and visualization using UMAP. Clusters were identified using a resolution of 0.4 and the Leiden algorithm for the integrated datasets. Cell types were assigned to each cell based on their highest cell type module score created from wild-type controls (described below).

scRNA-seq cell type identification and modules

For embryonic retinae, a representative E14.5 wild type dataset was used for cell type identification. The individual clusters defined with data processed using Harmony (see above) were evaluated for known markers of cell types present at E14.5 (see Table S1). Differentially expressed genes in each cell type (one or more clusters/cell type) were identified using the Seurat function FindAllMarkers with the default parameters. Then cell type module scores were created using genes with cluster-specific expression. Specifically, the top fifty genes for each cell type, after filtering (average natural log fold change greater than or equal to one, p-value of less than or equal to 0.001, expression in less than or equal to twenty percent of all other cell types), were used to generate the E14.5 retinal cell-type modules that were then used across all E14.5 datasets for individual cell type assignments.

For adult retinae, three representative wild type adult retina dataset was used for cell type identification. The individual clusters defined with data processed using Harmony (see above) were evaluated for known markers of cell types present in the adult mouse eye (see Table S1). Differentially expressed genes in each cell type (one or more clusters/cell type) were identified using the Seurat function FindAllMarkers with the default parameters. Then cell type module scores were created using genes with cluster-specific expression. Specifically, the top fifty genes for each cell type, after filtering (average natural log fold change greater than or equal to one, p-value of less than or equal to 0.001, expression in less than or equal to twenty percent of all other cell types), were used to generate the adult retinal cell-type modules that were then used across all datasets for individual cell type assignments.

Single cell ATAC-seq (scATAC-seq) data analysis

ScATAC-seq data were aligned to the mouse reference genome mm10 using the 10x Genomics Cell Ranger ATAC pipeline (v1.1.0) to call peaks and generate count matrices. The count matrices were then analyzed using Signac (v1.0.0, https://github.com/timoast/signac). In each dataset, cells were removed if: (1) the fraction of fragments in peaks < 15%, (2) the ratio of mono-nucleosomal to nucleosome-free fragments > 10, (3) the number or fragments in peak regions < 500 or > 100000, (4) the transcriptional start site (TSS) enrichment score < 2, and (5) the ratio of fragments in genomic blacklist regions > 0.05[19]. The retained data were normalized using the term frequency-inverse document frequency (TF-IDF) normalization method, followed by singular value decomposition (SVD) for dimensionality reduction and clustering. Clustering results were visualized using t-distributed stochastic neighbor embedding (t-SNE).

Cicero was used to calculate activity scores of genes based on accessibility of promoters and their co-accessible genomic regions[20]. To identify cell types, scATAC-seq data was integrated with scRNA-seq data based on the correlation between the gene activity estimated by Cicero and the transcriptomic expression pattern in the same tissue at the same time point. Cells in scATAC-seq datasets were classified to specific cell types using the integration method in Seurat[21], and only cells with a prediction score > 0.5 were included in the downstream analysis. To visualize the genomic accessibility within a specific region, signals from all cells in each cell type were averaged, and plots of tracks were generated using the CoveragePlot function in Signac.

Chromatin Immunoprecipitation

Freshly isolated retinae from 10 week old *Vsx2SE DoubleConsensus*$^{\Delta/\Delta}$ mice were isolated and immediately placed on dry ice. The frozen mouse retinae tissue was sent to Active Motif Services (Carlsbad, CA) to be processed for ChIP-Seq. In brief, tissue was immersed in PBS + 1% formaldehyde and incubated at room temperature for 15 minutes. Fixation was quenched by the addition of 0.125 M glycine (final) followed by two wash steps with PBS. Chromatin was isolated by the addition of lysis buffer, followed by disruption with a Dounce homogenizer. Lysates were sonicated and the DNA sheared to an average length of 300-500 bp. Genomic DNA (Input) was prepared by treating aliquots of chromatin with RNase, proteinase K and heat for de-crosslinking, followed by ethanol precipitation. Pellets were resuspended and the resulting DNA was quantified using a ClarioStar spectrophotometer. Extrapolation to the original chromatin volume allowed quantitation of the total chromatin yield.

An aliquot of chromatin (40 ug) was precleared with protein G agarose beads (Invitrogen). Genomic DNA regions of interest were isolated using 8 ul each of two antibodies against Vsx2 (Exalpha Biologicals Inc., catalog number X1179P, X1180P). Complexes were washed, eluted from the beads with SDS buffer, and subjected to RNase and proteinase K treatment. Crosslinks were reversed by incubation overnight at 65 °C, and ChIP DNA was purified by phenol-chloroform extraction and ethanol precipitation.

Quantitative PCR (qPCR) reactions were carried out in triplicate on specific genomic regions using SYBR Green Supermix (Bio-Rad, Cat # 170-8882) on a CFX Connect™ Real Time PCR

system. The resulting signals were normalized for primer efficiency by carrying out qPCR for each primer pair using Input DNA extracted from the cells.

Illumina sequencing libraries were prepared from the ChIP and Input DNAs by the standard consecutive enzymatic steps of end-polishing, dA-addition, and adaptor ligation. Steps were performed on an automated system (Apollo 342, Wafergen Biosystems/Takara). After a final PCR amplification step, the resulting DNA libraries were quantified and sequenced at the core facility at St Jude.

Vision Testing

The OptoMotry system from CerebralMechanics was used to measure the optomotor response of the CRISPR gene-edited mice. Briefly, a rotating cylinder covered with a vertical sine wave grating was calculated and drawn in virtual three-dimensional (3-D) space on four computer monitors facing to form a square. CRISPR gene-edited mice standing unrestrained on a platform in the center of the square tracked the grating with reflexive head and neck movements. The spatial frequency of the grating was clamped at the viewing position by repeatedly recentering the cylinder on the head. Acuity was quantified by increasing the spatial frequency of the grating until an optomotor response could not be elicited. Contrast sensitivity was measured at spatial frequencies between 0.1 and 0.45 cyc/deg.

In total, 3 *Region0*$^{\Delta/\Delta}$, 3 *Region0+1*$^{\Delta/\Delta}$, 3 *Region1*$^{\Delta/\Delta}$, 3 *Region2*$^{\Delta/\Delta}$, 3 *Region3*$^{\Delta/\Delta}$, 3 *DoubleConsensus*$^{\Delta/\Delta}$, 3 *ConsensusB*$^{\Delta/\Delta}$, and 3 *Vsx2 SE*$^{\Delta/\Delta}$ were analyzed along with a wildtype littermate for each region deletion. The tester was blinded to genotype until after testing was complete. Bar plot displays mean with SEM.

ChIP-seq data analysis

The ChIP-seq data were analyzed as described previously[22]. Briefly, low-quality base calls (Phred < 20) and adapter sequences were trimmed from the raw ChIP-seq reads using TrimGalore (v0.6.3). The trimmed reads were then mapped to the reference genome (mm10) using the Burrows-Wheeler Aligner (0.7.17-r1198 [23]). PCR duplicates were marked using the "bamsormadup" command from Biobambam2 (v.2.0.87 [24]). Next, the non-duplicated uniquely-mapped read pairs were extracted using SAMtools (v.1.9 [25]) with parameters -F 1048, -q 1. The fragment size were estimated from the uniquely mapped reads using cross correlation analysis by SPP (v1.1 [26]). Reads were then extended to the estimated fragment size and the genomic coverage was calculated using the 'genomecov' command from BEDTools (v.2.24.0 [9]). The bedGraphToBigWig tool from UCSC tools (v.4 [27]) was used to generated bigwig tracks normalized to 15 million uniquely mapped reads. Sharp peaks were called against the corresponding input sample using Macs2 (v 2.1.1.20160309 [28]) with parameters -g mm10 --nomodel --extsize -q 0.05. Regions showing five- to fifty- fold high-confidence enrichment ration to background were used to build the model. We identified 105,803 peaks for abX1179P and 86,438 peaks for abX1180P with FDR < 0.05.

References

1 Sentmanat, M. F., Peters, S. T., Florian, C. P., Connelly, J. P. & Pruett-Miller, S. M. A Survey of Validation Strategies for CRISPR-Cas9 Editing. *Scientific Reports* **8**, 888, doi:10.1038/s41598-018-19441-8 (2018).

2 Dhingra, A. *et al.* Probing neurochemical structure and function of retinal ON bipolar cells with a transgenic mouse. *J Comp Neurol* **510**, 484-496, doi:10.1002/cne.21807 (2008).

3 Huckfeldt, R. M. *et al.* Transient neurites of retinal horizontal cells exhibit columnar tiling via homotypic interactions. *Nat Neurosci* **12**, 35-43, doi:10.1038/nn.2236 (2009).

4 Siegert, S. *et al.* Genetic address book for retinal cell types. *Nat Neurosci* **12**, 1197-1204, doi:10.1038/nn.2370 (2009).

5 Vázquez-Chona, F. R., Clark, A. M. & Levine, E. M. Rlbp1 promoter drives robust Müller glial GFP expression in transgenic mice. *Invest Ophthalmol Vis Sci* **50**, 3996-4003, doi:10.1167/iovs.08-3189 (2009).

6 Dyer, M. A. & Cepko, C. L. The p57Kip2 cyclin kinase inhibitor is expressed by a restricted set of amacrine cells in the rodent retina. *J Comp Neurol* **429**, 601-614 (2001).

7 Dyer, M. A. & Cepko, C. L. p27Kip1 and p57Kip2 regulate proliferation in distinct retinal progenitor cell populations. *J Neurosci* **21**, 4259-4271, doi:10.1523/jneurosci.21-12-04259.2001 (2001).

8 Dyer, M. A., Livesey, F. J., Cepko, C. L. & Oliver, G. Prox1 function controls progenitor cell proliferation and horizontal cell genesis in the mammalian retina. *Nat Genet* **34**, 53-58, doi:10.1038/ng1144 (2003).

9 Quinlan, A. R. & Hall, I. M. BEDTools: a flexible suite of utilities for comparing genomic features. *Bioinformatics* **26**, 841-842, doi:10.1093/bioinformatics/btq033 (2010).

10 Wingender, E., Dietze, P., Karas, H. & Knüppel, R. TRANSFAC: a database on transcription factors and their DNA binding sites. *Nucleic Acids Res* **24**, 238-241, doi:10.1093/nar/24.1.238 (1996).

11 Bailey, T. L. *et al.* MEME SUITE: tools for motif discovery and searching. *Nucleic Acids Res* **37**, W202-208, doi:10.1093/nar/gkp335 (2009).

12 Kim, D. S., Matsuda, T. & Cepko, C. L. A core paired-type and POU homeodomain-containing transcription factor program drives retinal bipolar cell gene expression. *J Neurosci* **28**, 7748-7764, doi:10.1523/jneurosci.0397-08.2008 (2008).

13 Donovan, S. L. & Dyer, M. A. Preparation and square wave electroporation of retinal explant cultures. *Nature Protocols* **1**, 2710-2718, doi:10.1038/nprot.2006.454 (2006).

14 Goodson, N. B., Kaufman, M. A., Park, K. U. & Brzezinski, J. A. t. Simultaneous deletion of Prdm1 and Vsx2 enhancers in the retina alters photoreceptor and bipolar cell fate specification, yet differs from deleting both genes. *Development* **147**, doi:10.1242/dev.190272 (2020).

15 Bone-Larson, C. *et al.* Partial rescue of the ocular retardation phenotype by genetic modifiers. *J Neurobiol* **42**, 232-247, doi:10.1002/(sici)1097-4695(20000205)42:2<232::aid-neu7>3.0.co;2-4 (2000).

16 Zheng, G. X. Y. *et al.* Massively parallel digital transcriptional profiling of single cells. *Nature Communications* **8**, 14049, doi:10.1038/ncomms14049 (2017).

17 Hao, Y. *et al.* Integrated analysis of multimodal single-cell data. *Cell* **184**, 3573-3587.e3529, doi:10.1016/j.cell.2021.04.048 (2021).
18 Korsunsky, I. *et al.* Fast, sensitive and accurate integration of single-cell data with Harmony. *Nature Methods* **16**, 1289-1296, doi:10.1038/s41592-019-0619-0 (2019).
19 Amemiya, H. M., Kundaje, A. & Boyle, A. P. The ENCODE blacklist: identification of problematic regions of the genome. *Scientific reports* **9**, 1-5 (2019).
20 Pliner, H. A. *et al.* Cicero predicts cis-regulatory DNA interactions from single-cell chromatin accessibility data. *Molecular cell* **71**, 858-871. e858 (2018).
21 Stuart, T. *et al.* Comprehensive integration of single-cell data. *Cell* **177**, 1888-1902. e1821 (2019).
22 Zeineldin, M. *et al.* MYCN amplification and ATRX mutations are incompatible in neuroblastoma. *Nat Commun* **11**, 913, doi:10.1038/s41467-020-14682-6 (2020).
23 Li, H. & Durbin, R. Fast and accurate short read alignment with Burrows-Wheeler transform. *Bioinformatics* **25**, 1754-1760, doi:10.1093/bioinformatics/btp324 (2009).
24 Tischler, G. & Leonard, S. biobambam: tools for read pair collation based algorithms on BAM files. *Source Code for Biology and Medicine* **9**, 1-18 (2014).
25 Li, H. *et al.* The Sequence Alignment/Map format and SAMtools. *Bioinformatics* **25**, 2078-2079, doi:10.1093/bioinformatics/btp352 (2009).
26 Kharchenko, P. V., Tolstorukov, M. Y. & Park, P. J. Design and analysis of ChIP-seq experiments for DNA-binding proteins. *Nat Biotechnol* **26**, 1351-1359, doi:10.1038/nbt.1508 (2008).
27 Kuhn, R. M., Haussler, D. & Kent, W. J. The UCSC genome browser and associated tools. *Brief Bioinform* **14**, 144-161, doi:10.1093/bib/bbs038 (2013).
28 Zhang, Y. *et al.* Model-based analysis of ChIP-Seq (MACS). *Genome Biol* **9**, R137, doi:10.1186/gb-2008-9-9-r137 (2008).
