# Supplementary material for: Identification of a modular super-enhancer in murine retinal development
Source: Nat Commun. 2022 Jan 11;13:253. doi: 10.1038/s41467-021-27924-y (PMC8752785; doi:10.1038/s41467-021-27924-y)
Supplement: Supplementary file 2 — Description of Additional Supplementary Files [file 41467_2021_27924_MOESM2_ESM.docx]

Description of Additional Supplementary Files

File Name: Supplementary Dataset 1
Description: Genes used for single cell cell-type identification in scRNA-seq analysis. Rod depleted scRNA-seq analysis. Expression of bipolar genes in the WT and Vsx2 SE KO. Transcription factor motifs in the Vsx2 SE.

File Name: Supplementary Dataset 2
Description: RNA-seq of Vsx2 SE deletion strains (FPKM).

File Name: Supplementary Dataset 3
Description: qRT-PCR data of adult and E14.5 SE deletion retinae.

File Name: Supplementary Dataset 4
Description: EdU Scoring of Vsx2-SE-Region 1 retina at E14.5, E17.5, and P0.

File Name: Supplementary Dataset 5
Description: scRNA-seq cell type distribution for individual mouse strains with conserved module deletions.
