# Supplementary material for: Identification of a modular super-enhancer in murine retinal development
Source: Nat Commun. 2022 Jan 11;13:253. doi: 10.1038/s41467-021-27924-y (PMC8752785; doi:10.1038/s41467-021-27924-y)
Supplement: Supplementary file 8 — Reporting Summary [file 41467_2021_27924_MOESM8_ESM.pdf]

nature portfolio

Corresponding author(s): Michael Dyer

Last updated by author(s): 11/30/2021

Reporting Summary

Nature Portfolio wishes to improve the reproducibility of the work that we publish. This form provides structure for consistency and transparency in reporting. For further information on Nature Portfolio policies, see our Editorial Policies and the Editorial Policy Checklist.

Statistics

For all statistical analyses, confirm that the following items are present in the figure legend, table legend, main text, or Methods section.

n/a	Confirmed	
☐	☒	The exact sample size (*n*) for each experimental group/condition, given as a discrete number and unit of measurement
☐	☒	A statement on whether measurements were taken from distinct samples or whether the same sample was measured repeatedly
☐	☒	The statistical test(s) used AND whether they are one- or two-sided<br>*Only common tests should be described solely by name; describe more complex techniques in the Methods section.*
☐	☒	A description of all covariates tested
☐	☒	A description of any assumptions or corrections, such as tests of normality and adjustment for multiple comparisons
☐	☒	A full description of the statistical parameters including central tendency (e.g. means) or other basic estimates (e.g. regression coefficient) AND variation (e.g. standard deviation) or associated estimates of uncertainty (e.g. confidence intervals)
☐	☒	For null hypothesis testing, the test statistic (e.g. *F*, *t*, *r*) with confidence intervals, effect sizes, degrees of freedom and *P* value noted<br>*Give P values as exact values whenever suitable.*
☒	☐	For Bayesian analysis, information on the choice of priors and Markov chain Monte Carlo settings
☒	☐	For hierarchical and complex designs, identification of the appropriate level for tests and full reporting of outcomes
☐	☒	Estimates of effect sizes (e.g. Cohen's *d*, Pearson's *r*), indicating how they were calculated

Our web collection on statistics for biologists contains articles on many of the points above.

Software and code

Policy information about availability of computer code

Data collection	Code used to process all data can be found at https://github.com/CodyRamirez/StJude_Scripts/tree/main/Vsx2_SE under the MIT license.
Data analysis	Sequencing reads were processed using 10X Genomics Cell Ranger version 6.0.016, with reads mapping to the mouse reference genome mm10 version3.0.0 (10x Genomics). Quality control (QC) filtering, clustering, dimensionality reduction, visualization, and differential gene expression were performed using Seurat v4.0.417 with R v4.1.0.<br>Code used to process all data can be found at https://github.com/CodyRamirez/StJude_Scripts/tree/main/Vsx2_SE under the MIT license.

For manuscripts utilizing custom algorithms or software that are central to the research but not yet described in published literature, software must be made available to editors and reviewers. We strongly encourage code deposition in a community repository (e.g. GitHub). See the Nature Portfolio guidelines for submitting code & software for further information.

Data

Policy information about availability of data

All manuscripts must include a data availability statement. This statement should provide the following information, where applicable:

- Accession codes, unique identifiers, or web links for publicly available datasets
- A description of any restrictions on data availability
- For clinical datasets or third party data, please ensure that the statement adheres to our policy

The sequencing data generated in this study have been deposited in the GEO database under accession code GSE169262 and reviewer token wfqtumeetbszhsp. Data is publicly available. Reference genome mm10 has been used for sequencing data.

Field-specific reporting

Please select the one below that is the best fit for your research. If you are not sure, read the appropriate sections before making your selection.

☒ Life sciences ☐ Behavioural & social sciences ☐ Ecological, evolutionary & environmental sciences

For a reference copy of the document with all sections, see nature.com/documents/nr-reporting-summary-flat.pdf

Life sciences study design

All studies must disclose on these points even when the disclosure is negative.

Sample size	Generally, litttermates were used and multiple animals of each genotype were analyzed. For Fig 1e, two mice totaling 4 retinae for each condition was analyzed with the exception of Grm6-GFP SEKO in which 3 retinae were analyzed. In Figure 1g, 4 retinae for each condition were analyzed. In 2b, e and f, we analyzed the entire litter resulting from a heterozygous cross and therefore the number of pups analyzed for each genotype was based on a Mendelian ratio. When n<3, statistical significance and error bars are not included. In Fig. 4l, an entire wild-type litter was electroporated at P0 for each construct, and at P21 three pups were randomly selected for analysis. In Fig. 5e, 3 mice for each deletion strain were selected for vision testing.
Data exclusions	Data has not been excluded.
Replication	Multiple techniques were used to validate the same findings including bulk RNA seq, scRNA seq, and qRT-PCR. There have been no instances in which repeat experiments yielded conflicting results, suggesting reproducibility of experiments.
Randomization	Animals of the appropriate age were selected at random.
Blinding	Data was analyzed prior to genotyping the animals when possible. Investigators were blinded to genotype or plasmid when scoring images. This is applicable for experiments in Figures 1g, 1i, 2b, 2e, 2f, 2n, 2o, 2p, 4l, and Supplemental Figures 1e-g. The Optomotry assay was blinded when vision testing mice for Figure 5e.

Reporting for specific materials, systems and methods

We require information from authors about some types of materials, experimental systems and methods used in many studies. Here, indicate whether each material, system or method listed is relevant to your study. If you are not sure if a list item applies to your research, read the appropriate section before selecting a response.

Materials & experimental systems

n/a	Involved in the study	
☐	☒	Antibodies
☐	☒	Eukaryotic cell lines
☒	☐	Palaeontology and archaeology
☐	☒	Animals and other organisms
☒	☐	Human research participants
☒	☐	Clinical data
☒	☐	Dual use research of concern

Methods

n/a	Involved in the study	
☐	☒	ChIP-seq
☐	☒	Flow cytometry
☒	☐	MRI-based neuroimaging

Antibodies

Antibodies used	rabbit anti-GFP (Invitrogen, catalog number A21311) 1:500<br>Clone M4, mouse anti-mouse, PKC-a (Upstate, catalog number 05-154) 1:5000<br>sheep anti-mouse, Vsx2 (Exalpha, catalog number X1180P) 1:200<br>sheep anti-mouse Vsx2 (Exalpha, catalog number X1179P) 1:200<br>rabbit anti-mouse Caspase-3 (BD Pharmingen, catalog number 559565) 1:1000
Validation	All antibodies have been routinely used to identify retinal cells in retina.<br>Hiler D, Chen X, Hazen J, Kupriyanov S, Carroll PA, Qu C, Xu B, Johnson D, Griffiths L, Frase S, Rodriguez AR, Martin G, Zhang J, Jeon J, Fan Y, Finkelstein D, Eisenman RN, Baldwin K, Dyer MA. Quantification of Retinogenesis in 3D Cultures Reveals Epigenetic Memory and Higher Efficiency in iPSCs Derived from Rod Photoreceptors. Cell Stem Cell. 2015 Jul 2;17(1):101-15. doi: 10.1016/j.stem.2015.05.015. PMID: 26140606; PMCID: PMC4547539.

Eukaryotic cell lines

Policy information about cell lines

Cell line source(s)	Neuro-2a cells (ATCC, Cat. #CCL-131)
Authentication	Cell lines were purchased from ATCC and authenticated in house by Powerplex.
Mycoplasma contamination	This cell line tested negative for mycoplasma contamination.
Commonly misidentified lines (See ICLAC register)	N/A

Animals and other organisms

Policy information about studies involving animals; ARRIVE guidelines recommended for reporting animal research

Laboratory animals	Mus musculus, C57BL/6J and 129S1/Sv-Vsx2or-J/J, adult male and female. Mice are kept on a 12 hour light/dark schedule and fed ad libitum. The humidity of the atrium is maintained between 40 and 60%.
Wild animals	This study did not involve wild animals.
Field-collected samples	No field-collected samples.
Ethics oversight	All animal procedures and protocols were approved by the St. Jude Laboratory Animal Care and Use Committee. All studies conform to federal and local regulatory standards.

Note that full information on the approval of the study protocol must also be provided in the manuscript.

ChIP-seq

Data deposition

☒ Confirm that both raw and final processed data have been deposited in a public database such as GEO.

☒ Confirm that you have deposited or provided access to graph files (e.g. BED files) for the called peaks.

Data access links *May remain private before publication.*	Data is publicly available on GEO and mapped to reference genome mm10. GSE169262.
Files in database submission	bulk RNA-seq, scRNA-seq, scATAC-seq, ChIP-seq
Genome browser session (e.g. UCSC)	No longer applicable.

Methodology

Replicates	Two retinae from a 10 week old mouse were isolated. Quantitative PCR (qPCR) reactions were carried out in triplicate on specific genomic regions using SYBR Green Supermix (Bio-Rad, Cat # 170-8882) on a CFX Connect™ Real Time PCR system. The resulting signals were normalized for primer efficiency by carrying out qPCR for each primer pair using Input DNA extracted from the cells.
Sequencing depth	Total number of reads per sample: 50,000,000
Antibodies	Vsx2, Exalpha Biologicals Inc., catalog number X1179P and X1180P
Peak calling parameters	Sharp peaks were called against the corresponding input sample using Macs2 (v 2.1.1.20160309; PMID: 18798982) with parameters -g mm10 --nomodel --extsize -q 0.05.
Data quality	Regions showing five- to fifty- fold high-confidence enrichment ration to background were used to build the model. We identified 105,803 peaks for abX1179P and 86,438 peaks for abX1180P with FDR < 0.05.
Software	The ChIP-seq data were analyzed as described previously (PMID: 32060267). Briefly, lowquality base calls (Phred < 20) and adapter sequences were trimmed from the raw ChIP-seq reads using TrimGalore (v0.6.3). The trimmed reads were then mapped to the reference genome (mm10) using the Burrows-Wheeler Aligner (0.7.17-r1198, PMID: 19451168). PCR duplicates were marked using the "bamsormadup" command from Biobambam2 (v.2.0.87, PMC4075596). The non-duplicated uniquely-mapped read pairs were extracted using SAMtools (v.1.9, PMID: 19505943) with parameters -F 1048, -q 1. The fragment size were estimated from the uniquely mapped reads using cross correlation analysis by SPP (v1.1, PMID: 19029915). Reads were then extended to the estimated fragment size and the genomic coverage was calculated using the 'genomecov' command from BEDTools (v.2.24.0, PMID: 20110278). The bedGraphToBigWig tool from UCSC tools (v.4, PMID: 22908213) was used to generated bigwig tracks normalized to 15 million uniquely mapped reads. Sharp peaks were called against the corresponding input sample using Macs2 (v 2.1.1.20160309; PMID: 18798982) with parameters -g mm10 --nomodel --extsize -q 0.05.

Flow Cytometry

Plots

Confirm that:

- [x] The axis labels state the marker and fluorochrome used (e.g. CD4-FITC).
- [x] The axis scales are clearly visible. Include numbers along axes only for bottom left plot of group (a 'group' is an analysis of identical markers).
- [x] All plots are contour plots with outliers or pseudocolor plots.
- [x] A numerical value for number of cells or percentage (with statistics) is provided.

Methodology

Sample preparation	Retina dissociation buffer was prepared by adding 40 U papain (Worthington CAT#LS003119) to 400 uL of papain buffer and incubating at 37°C for 15 min. Retinas were individually dissected in retinal explant media (REM) and placed on ice. Four hundred microliters of buffer was added to each retina and incubated at 37°C. To dissociate the retina, tubes were agitated twice at 5 min intervals and 40 uL of DNAse solution (DS) was added and incubated at 37°C for an additional 5 min. The cell suspension was filtered through a 40-µm cell strainer (Falcon CAT#352340) and the filter was washed with PBS to bring the total volume to 1.4 mL.
Instrument	FACS Aria Fusion (Becton Dickinson)
Software	Diva software (Becton Dickinson)
Cell population abundance	DAPI was used to identify dead cells and scatter was used to determine debris. Live cells accounted for over 70% of events. We sorted for GFP+ cells. All retinae contain GFP+ cells except for the Grm6-GFP CRC-SE knockout (Source Data). The percentage of GFP+ cells for each retina was determined by the number of GFP+ events divided by all Live Cell events.
Gating strategy	Cell death was assessed using a forward scatter versus DAPI plot. GFP+ cells were assessed.

- [x] Tick this box to confirm that a figure exemplifying the gating strategy is provided in the Supplementary Information.
